# Heterozygosity increases microsatellite mutation rate, linking it to demographic history

**DOI:** 10.1186/1471-2156-9-72

**Published:** 2008-11-14

**Authors:** William Amos, Jonathan Flint, Xin Xu

**Affiliations:** 1 Department of Zoology, Downing Street, Cambridge, CB4 3DB, UK; 2 Wellcome Trust Centre for Human Genetics, Roosevelt Drive, Oxford, OX3 7BN, UK; 3 Program for Population Genetics, Harvard School of Public Health, 665 Huntington Ave, Boston, MA 02115, USA

## Abstract

**Background:**

Biochemical experiments in yeast suggest a possible mechanism that would cause heterozygous sites to mutate faster than equivalent homozygous sites. If such a process operates, it could undermine a key assumption at the core of population genetic theory, namely that mutation rate and population size are indpendent, because population expansion would increase heterozygosity that in turn would increase mutation rate. Here we test this hypothesis using both direct counting of microsatellite mutations in human pedigrees and an analysis of the relationship between microsatellite length and patterns of demographically-induced variation in heterozygosity.

**Results:**

We find that microsatellite alleles of any given length are more likely to mutate when their homologue is unusually different in length. Furthermore, microsatellite lengths in human populations do not vary randomly, but instead exhibit highly predictable trends with both distance from Africa, a surrogate measure of genome-wide heterozygosity, and modern population size. This predictability remains even after statistically controlling for non-independence due to shared ancestry among populations.

**Conclusion:**

Our results reveal patterns that are unexpected under classical population genetic theory, where no mechanism exists capable of linking allele length to extrinsic variables such as geography or population size. However, the predictability of microsatellite length is consistent with heterozygote instability and suggest that this has an important impact on microsatellite evolution. Whether similar processes impact on single nucleotide polymorphisms remains unclear.

## Background

One of the most commonly encountered terms in classical population genetic theory is the compound quantity N_e_μ, where N_e _is the effective population size and μis the mutation rate. By implication, these two terms are assumed to be independent, an assumption that has never seriously been challenged. However, it has been suggested that heterozygous sites might be more mutable than equivalent homozygous sites [1], potentially linking evolutionary rate to demography, with changes in population size feeding back to mutation rate through changes in heterozygosity. Evidence for heterozygote instability (HI) has been largely anecdotal [1] and disputed [2-4]. If HI does influence mutation rate, its effects are likely to be most easily detected in fast-evolving sequences such as microsatellites.

Microsatellites are abundant, highly polymorphic DNA sequences that evolve mainly through the slippage-mediated gain and loss of single repeat units [5,6]. However, there are a range of additional elements such as mutation bias [1,7], length dependent mutation rates [8], some as yet unresolved limit to maximum repeat number (often called length boundaries) [9,10], mutations that reduce slippage by interrupting the repeat tract [11] and occasional multi-step mutations [12]. These variables have led to many alternative models of microsatellite evolution, but all share the same expectation that every locus will vary independently in length over time [13], with every microsatellite in every population being as likely to be longer than average as it is to be shorter. Consequently, the observation that maize microsatellite length varies predictably with altitude [14] is unexpected and perhaps indicates some important aspect of microsatellite evolution has thus far been overlooked.

Explaining trends in mean allele length across a large number of independent markers sampled from populations within a species is not easy. The most obvious possibility is natural selection, but this seems unlikely because of the vast number of markers involved. Not only is it unclear how slightly greater length at one particular (non-genic) microsatellite allele would impact on fitness, but also, even if there was an influence on fitness, the large number of segregating loci would mean that the differential fitness between individuals would likely be negligible. An alternative possibility might involve genes associated with DNA replication or mismatch repair. If such genes are polymorphic, carrying alleles that increase or decrease the genome-wide microsatellite mutation rate, a biased mutation process will tend to generate predictable differences in mean allele length. For example, among microsatellites showing an upward mutation bias, a population carrying a high mutation rate allele would, over time, carry longer microsatellites compared with a related population in which a slow mutation rate allele was common. Mutator alleles of this kind are known [15,16], but are generally associated with cancer and hence may be rare in natural populations.

A third possible mechanism with the potential to drive differences in microsatellite length between populations is based on the idea of heterozygote instability. In yeast, elegant molecular studies have shown that, when homologous chromosomes pair during meiosis, heterozygous sites are recognised and 'repaired' by gene conversion-like events [17]. This extra round of DNA synthesis could in principle provide an extra opportunity for slippage-generated mutations at heterozygous compared with equivalent homozygous sites. If so, and if similar processes operate in higher organisms, a link could exist between demography and microsatellite mutation rate. For example, if a population expands, genome-wide heterozygosity would tend to increase which in turn would feed back to increase the genome-wide mutation rate.

The heterozygote instability model is currently speculative, but in principle it could explain how maize microsatellite length could vary with altitude. For example, if the effective population size varies with altitude this would generate a gradient in heterozygosity which in turn would create a gradient in mutation rate. As with mutator alleles, in the presence of a directional mutation bias [1,18], variation in mutation rate among populations will tend to drive predictable variation in mean allele length. Here we attempt to test the HI hypothesis in two ways. First we use a direct analysis of mutations identified in human pedigrees to ask whether mutations occur preferentially in heterozygous individuals. Second, we ask whether microsatellite length and heterozygosity are correlated by exploiting the well-documented trend for human heterozygosity to decrease with distance from Africa.

## Results

### Pedigree mutation analysis

**Figure 1:**
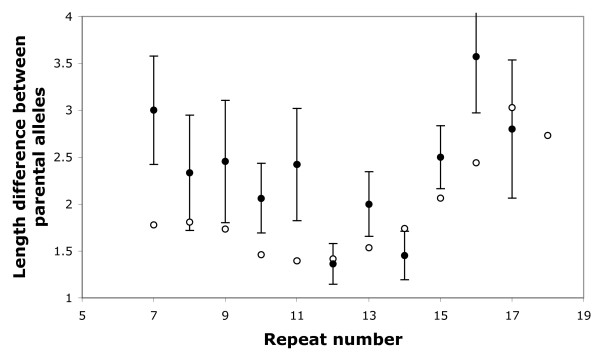
**Influence of genotype span on mutation rate**. Span is defined as the difference in allele length, measured in repeat units, of alleles in the parental genotype in which a mutation was identified. For each mutation, the expected span was calculated by averaging across all other alleles of that length in the dataset (open circles). Data from 203 observed mutations are presented as filled circles ± 1 standard error. For clarity, the graph is truncated to include only repeat numbers where three or more mutations were recorded, thereby excluding 12 of the original 215 mutations (span range 0 – 16). Considering all 215 mutations, mean span of mutated alleles is significantly greater than expected by chance (one sample t-test, *t*_ [214] _= 3.02, p = 0.0028, two-tailed).

As a direct test of HI we reanalysed 256 mutations identified in tetranucleotide repeat markers in a genome screen for hypertension [19,20]. To control for the tendency for longer alleles to be both more mutable and associated with larger 'span' (defined as the difference in length between alleles in the parental genotype), we combined published clone and primer sequences to convert raw allele lengths into repeat numbers. We then compared the span of mutated alleles with the mean span of all alleles in the dataset carrying the same number of repeats (Figure 1). On average, mutated alleles occur in genotypes with significantly greater span than expected by chance (one sample t-test, mean excess span = 0.46 repeats, *t*_ [214] _= 3.02, p = 0.0028, two-tailed).

### Predictability of microsatellite length across populations

To test the possible impact of heterozygote instability on microsatellites in real populations requires a series of populations that vary in their genome-wide heterozygosity due to clearly established differences in demographic history. Humans provide just such an opportunity because as we moved out of Africa to colonise the world, population bottleneck(s) eroded genetic variability [21]. The result is a smooth decline in variability with distance from Africa, observed similarly in single nucleotide polymorphisms [22], microsatellites [23], morphological traits [24] and even in the diversity of commensal bacteria [25]. Under HI, this gradient in heterozygosity should create a parallel gradient in relative genome-wide mutation rate. Although mutation rate is difficult to measure directly, there is widespread evidence that many, and possibly most, microsatellites are prone to mutation bias [1,14,18,26,27] whereby gains in length outnumber losses or vice verse. When present, such biases have the potential to translate differences in mutation rate between populations into differences in relative mean allele length.

To test the HI hypothesis, we used general linear models (GLMs) to look for a correlation between human microsatellite length and distance from Africa (='distance'), using a large, published dataset of 783 microsatellites genotyped in the human diversity panel of 1048 individuals from 53 worldwide populations [28,29]. In addition to distance, we considered three further predictor variables. First, it has been reported that the direction of bias sometimes reverses, probably when a microsatellite reaches some maximum length [10,19], potentially creating non-linearities that might be missed by a simple linear regression of allele length on distance. Consequently, we also fitted distance from Africa squared. Second, following the initial colonisation of the world, some populations have remained small or even declined, while others have expanded massively. To capture something of these more recent trends we also fitted log modern population size (= "size"). Finally, given that the direction of mutation bias will determine whether a mutation rate increase results in relatively longer or shorter microsatellites, we also include allele length skew. Skew is a weak predictor of the direction of bias [30] but is also expected to be correlated with mean allele because it reflects the relative frequencies of short and long alleles.

One approach would be to fit all data together in a single GLM, with 'locus' fitted either as a random or fixed factor. However, a proportion of loci will inevitably behave aberrantly due variously to deletions or insertions in the flanking sequence, to large 'jump' mutations or to heterogeneity of slippage rate among alleles caused by interruption mutations. Even if such loci are rare, they could reduce greatly the fit of the model. In addition, the dataset includes di-, tri- and tetranuleotide repeats, adding additional heterogeneity. Consequently, we decided to fit separate GLMs for each marker in turn, using mean repeat number as the response variable and skew, size, distance, distance squared and all second order interactions as predictors. Each model was then simplified by backward stepwise elimination, aimed at minimising the Akaike Information Criterion (AIC), to achieve the minimum adequate model (MAM). AIC is used for convenience when fitting large numbers of models, and we acknowledge that this is by no means a perfect measure and may somewhat inflate significance.

**Figure 2:**
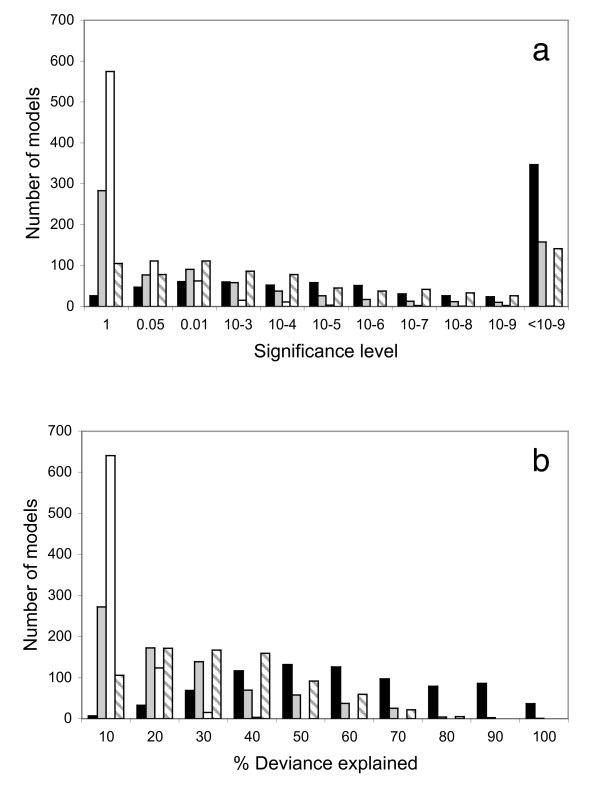
**To what extent can variation in human microsatellite length be predicted by demographically induced variation in heterozygosity and allele length skew?**. Separate general linear models were fitted to data from each of 783 microsatellites genotyped in 53 worldwide populations, with mean allele length as the response and distance to Africa, distance from Africa squared, skew in allele length and log(modern population size) plus all second order interactions as predictor variables. Distance from Africa is taken as the land-only distance from Addis Ababa in kilometres [22,23]. Data are from [29]. Each model was simplified by backward elimination to achieve the minimum adequate model, MAM. The significance of the three primary terms (skew, log population size, distance + distance^2^) were then estimated by dropping each in turn and using ANOVA to compare the resulting MAM with the MAM produced when all terms were fitted. Figure 2a: number of models achieving a given level of significance for the full models (black bars) and for dropping skew (grey bars), population size (white bars) or distance + distance^2 ^(striped bars). Values on the X axis refer to lower bin boundary; i.e. '1' indicates non-significant models with P > 0.05, '0.05' indicates models with P-values lying between 0.05 and 0.01. Figure 2b: number of models explaining a given proportion of the null deviance. Colour coding of the bars is the same as in Figure 2a but X axis values are upper bin boundaries

Across the 783 microsatellites examined, 757 (96.7%) explain significant amounts of variation in mean length across populations at α = 5%, and 578 models are significant after Bonferroni correction for the number of loci examined (α = 0.05/783 = 6.4 × 10^-5^). The MAMs explain an average of 54% of the null deviance. We then assessed the importance of each of our original predictor variables, refitting the models three times, excluding in turn size, skew and distance (plus distance squared, removed together). The significance of each of these three primary terms was tested by asking whether its exclusion significantly reduced the proportion of deviance explained compared with the equivalent full MAM where all three primary terms were fitted. Numbers of models achieving a given level of significance and explaining a given proportion of the null deviance are summarised in Figures 2a and 2b respectively. See Additional File 1 for a full list of all coefficients for terms retained in these MAMs. Distance (plus distance squared) contributes significantly to 678 of 783 models, and explains on average 28.1% of the null deviance across all models. Skew is expected *a priori *to correlate with mean allele length and contributes significantly to 500 models, explaining on average 21.1% of the null deviance. The second demographic term, population size, contributes significantly to 208 models at α = 5% (97 at α = 1%) and on average explains 5.1% of the null deviance. Although modest, the explanatory power of population size is commensurate with its contribution to the original regression of heterozygosity on distance from Africa (correlation between heterozygosity and distance from Africa alone, r^2 ^= 86% [23]; adding in log(modern population size) plus the interaction with heterozygosity increases the r^2 ^to 90%).

Our results suggest a high degree of predictability of mean allele length across human populations. However, neighbouring populations will tend to share a common origin which, together with migration, could create an autocorrelation in allele length that might inflate statistical significance. To control for the possibility of phylogenetic non-independence, we used the method of comparative analysis through independent contrasts, CAIC [31]. In this approach, the data set of N taxa is mapped onto a phylogeny from which are extracted N-1 comparisons between pairs of taxa that share a common node. Trait values are then expressed as absolute differences in trait value between the taxon-pairs, thereby removing the effect of shared ancestry by capturing only the variation in the trait that has occurred since each taxon-pair diverged. For our phylogeny we chose to use a neighbour-joining tree based on a similarity matrix of land-only geographic distances, thereby avoiding possible circularities that might arise if the tree was calculated from the same genetic data that were being analysed for trends in allele length. Use of genetic distances for the measure of population similarity yields essentially identical results.

**Figure 3:**
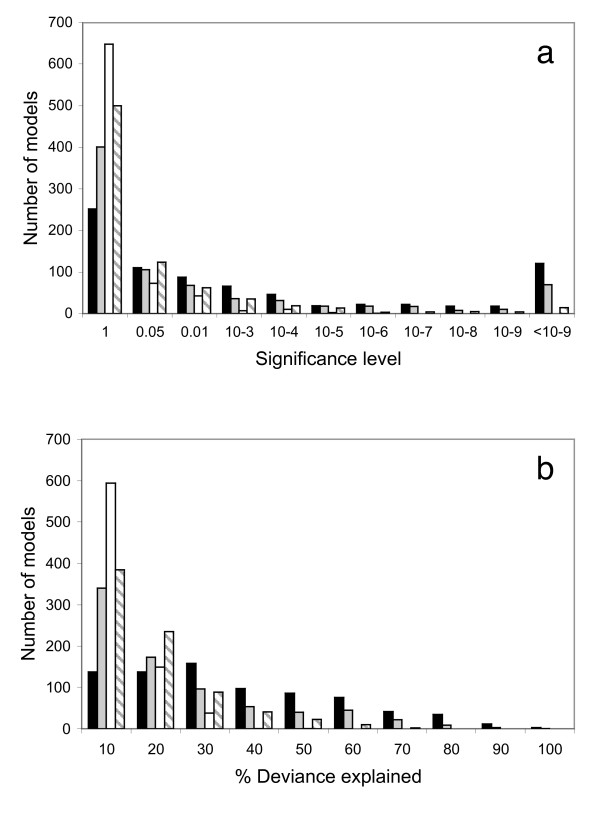
**To what extent can variation in human microsatellite length be predicted by demographically induced variation in heterozygosity and allele length skew after full phylogenetic correction?**. Repeat of the analysis presented in Figure 2 above, but this time with full correction for possible phylogenetic non-independence using the method of independent contrasts [31]. Briefly, the input data are replaced with absolute differences in trait value between the N-1 taxon pairs that share a common node, where N is the number of populations (53). Figure 3a: number of models achieving a given level of significance for the full models (black bars) and for dropping skew (grey bars), population size (white bars) or distance + distance^2 ^(striped bars). Values on the X axis refer to lower bin boundary; i.e. '1' indicates non-significant models with P > 0.05, '0.05' indicates models with P-values lying between 0.05 and 0.01. Figure 3b: number of models explaining a given proportion of the null deviance. Colour coding of the bars is the same as in Figure 3a but X axis values are upper bin boundaries.

Having extracted independent contrasts for the four primary predictor variables, we then refitted all models and repeated the full analysis described above, summarised in Figure 3. The coefficients for all terms retained in MAMs are presented in Supplementary Material Table 2. As before, a high proportion of models are significant (531 = 67.8%), explaining an average of 31.4% of the null deviance. Skew, population size and distance (plus distance squared) are significant in 382, 135 and 283 models respectively and on average explain 18.6%, 6.1% and 12.7% of the null deviance. See Additional File 2 for a full list of all coefficients for terms retained in these phylogenetically corrected MAMs.

Finally, although partitioning the predictors of genome-wide heterozygosity into their components, distance from Africa and population size, allows the relative contribution of each to be estimated, as well as any interactions with each other or with skew, there is a clear prediction that average heterozygosity alone should be a strong predictor of relative microsatellite length. To test this prediction we fitted multiple regressions to each locus in turn, with the response variable being mean repeat number in each population and predictor variables being average heterozygosity across all loci and average heterozygosity squared. A total of 568 of 783 models are significant at P < 0.05, the 783 models on average explaining 21% of the total variation in mean repeat number.

## Discussion

Here we conduct two tests of the heterozygote instability model, first examining human microsatellite mutations identified in pedigrees and then looking for a correlation between mean allele length and each human population's distance from Africa. We find that any given length of allele is more likely to mutate when it has a dissimilar length homologue and that mean allele length is strongly predicted by both modern population size and a surrogate measure of genome-wide heterozygosity, distance from Africa.

The greater mutability of alleles with a large span appears to support the heterozygote instability hypothesis. However, this only holds if the only (or primary) other determinant of mutation rate is repeat number. Whether this is true is open to debate. Within a repeat type (di-, tri- or tetranucleotide), repeat number is certainly a good predictor of apparent mutation rate [8,18,19,32] and other influences appear less important [32], but it is difficult to be sure that further factors have not been overlooked. If other factors are important such that loci exist with the same mean repeat number but substantially different mutation rates, mutations will occur preferentially at the more mutable locus and these loci will, as a result, also tend to have higher mean span. Controlling for such an effect would require a very large sample of mutations, much greater than the data set we have access to. Consequently, we sought indirect but arguably more powerful evidence from populations that exhibit well-established, demographically induced variation in heterozygosity.

A strong link between distance from Africa and microsatellite length is not expected under any of the standard neutral models of microsatellite evolution, but could conceivably arise without invoking HI in one of three ways. One possibility is natural selection, for example if longer alleles were favoured in warmer climates. A second possibility is ascertainment bias [3], whereby marker development tends to select for long microsatellites from a single population. However, both these alternative explanations make the same strong prediction that most loci will exhibit similar trends in terms of where in the world length is maximal and of the direction of the slope of the regression. For example, if ascertainment bias is important we would expect microsatellites to be consistently longer in Europe compared with elsewhere. In practice, different loci exhibit a wide diversity of trends, with mean allele length almost as likely to increase as to decrease with distance from Africa (linear regressions, 349 negative slopes compared with 434 positive slopes). In addition, ascertainment bias is expected to be negligible within a species [33] and predicts trends with a focus in Europe [3], when in reality the average trend has a focus in Africa [24,34]. Indeed, we confirmed the African origin by repeating the GLM analysis for each of all possible terrestrial origins across the world at 5 degree resolution. Averaged across all 783 loci, the greatest mean proportion of variation in allele length is explained if the origin is placed in southern Africa, and all non-African origins yield significantly weaker average trends (data not shown).

The third model for how markers across the genome could show correlated patterns would be if mutation rate or mutation bias were to be under the control of a polymorphic gene. Whilst difficult to exclude, this seems unlikely on several grounds. First, as with the previous two hypotheses, the expectation would be for all loci to behave similarly, rather than in the diverse patterns actually seen. Second, this mechanism seems unlikely to interact either with modern population size or, specifically, with distance from Africa (as opposed to any other geographic trend). Finally, this explanation does not include any obvious way to generate global trends rather than a patchwork of local differences between one region and another.

One further model for how to link microsatellite mutation rate to population size has been suggested by Ellegren [18]. He states that: "With allele frequency distributions being positively skewed, population contraction and bottlenecks will favour the survival of short, common alleles simply by random drift. Following such processes, the rate of microsatellite evolution should be expected to slow down because the intrinsic mutation rate of surviving alleles will be low." However, this argument appears false because it requires the mean of a series of random samples to differ from the mean of the parent distribution. To illustrate, consider the extreme case of a single locus in a series of populations so severely bottlenecked that each one carries only a single allele. With many such populations, the distribution of allele lengths will closely match the parent distribution. Consequently, while it is true that if the parent distribution is positively skewed a majority of populations will carry alleles that are shorter than the mean, the *average *length of surviving alleles will remain the same as that of the unbottlenecked parent population.

In contrast to the expectations of natural selection, ascertainment bias, a control gene or neutral drift, heterozygote instability appears to offer a plausible explanation for the correlations we observe. Distance from Africa and modern population size are strong and weak predictors of genome-wide heterozygosity respectively. Under HI, both these factors should act to modulate microsatellite mutation rate. In the absence of any mutation bias, the resulting variation in mutation rate between populations would impact only on the variance in allele length. However, almost all studies of microsatellite mutations report varying levels of mutation bias [1,14,18,26], such that any given locus is likely either to be progressively expanding or progressively contracting over time, with a strong suggestion that bias reverses at the upper length boundary [19]. When combined with a general trend for mutation rate to decrease with distance from Africa, this variation would tend to generate the patterns we see, where different microsatellites variously increase in length with distance from Africa, decrease in length or reveal rather humped distributions. Such patterns thus add weight both to the notion that most microsatellite mutations are biased, and to the possibility that bias can occasionally reverse.

Under heterozygote instability, a key assumption of population genetic theory appears to be false, at least for microsatellites. Instead of population size interacting with a fixed mutation rate to determine an equilibrium heterozygosity, our analysis suggests that a feedback loop can operate causing heterozygosity to increase over time, each increase also increasing the mutation rate which in turn raises heterozygosity. Within this framework, changes in population size may act to modulate the rate of change of heterozygosity, with population expansion causing acceleration and population contraction causing a slowdown. For example, population decline erodes genetic variability through drift, reducing heterozygosity and hence potentially lowering the genome-wide mutation rate relative to a related population that has not declined. Such effects have previously gone unnoticed but this is perhaps expected because the resulting differences in mean length are slight and most species do not exhibit the strong, well-defined trend in heterozygosity seen in humans. None the less, we show that the effects of HI can be far from trivial, with highly predictable trends in mean length developing over time spans of the order of 1000 generations.

Although we conclude that, among the alternative models considered in the paper, HI offers the most parsimonious explanation for the patterns we describe, determining the size of the effect and the exact way HI interacts with the other forces shaping microsatellite evolution will require further modelling. However, it is already clear that many standard analyses will give misleading results, since, in humans, microsatellites in populations equidistant from Africa will be more similar in length than in similarly spaced populations where one lies much closer to Africa than the other. Also, although it is unclear whether HI impacts on single nucleotide polymorphisms as well as microsatellites, there are reasons for believing this may be so. Thus, the studies in yeast suggest that single nucleotide polymorphisms are recognised while the chromosomes are in synapsis and attract 'repair' through gene conversion like events in which one strand is removed and resynthesised. Unless this process is perfect, DNA replication errors could plausibly cause new mutations to occur in the regions neighbouring the original SNP. However, without the directional evolution that affects microsatellites, formulating a convincing test is not easy. Finally, if HI is found to affect SNPs it might not simply be a quirk of evolution, but could even be adaptive. Specifically, HI would tend to attract mutations towards regions where polymorphism is already present, and hence tolerated or even beneficial, and away from monomorphic regions which will include those regions where selection has eliminated polymorphism. Consequently, it could be argued that HI would be beneficial and promoted by natural selection.

## Conclusion

In conclusion, the hypothesis that heterozygote sites could be more mutable than equivalent homozygous sites predicts that the length of human microsatellites in diverse populations will correlate with distance from Africa, responding to a strong gradient in genome-wide heterozygosity that arose due to one or several population bottlenecks that occurred as modern humans colonised the world. We find that microsatellite length is indeed highly predictable, varying both with distance from Africa and modern population size, a second correlate of heterozygosity. Alternative explanations for this predictability such as ascertainment bias or natural selection fail to account for the impact of population size and tend to require all loci to exhibit similar trends, when in fact a diversity of trends is seen. Consequently, we believe that heterozygote instability provides the most convincing explanation for the patterns we observe. The existence of heterozygote instability would challenge a key assumption of classical population genetic theory, namely the independence of population size and mutation rate, and would require a reappraisal of how genetic diversity originates and is maintained.

## Methods

### Mutation analysis

Mutation analysis was conducted on 236 tetranucleotide repeat marker mutations identified and verified previously in 287,786 parent-offspring transmissions in a genome-wide screen for genes associated with hypertension [19,20]. Of 273 markers, 33 were removed because of deviations from the expected 4-based periodicities or because of discrepancies between observed and expected product sizes (4 cases), leaving 215 mutations and 240 markers. At each locus, primer sequences were matched to the original clone sequence, thereby revealing the flanking sequence and the longest pure tract of repeats. Repeat number in all recorded alleles was then inferred by assuming the length of the flanking sequence was constant. Although this assumption may not hold in some cases, at the overwhelming majority of loci inferred repeat numbers fell within a consistent and plausible range (0 to 30 repeats). At a small number of loci, marginally negative repeat numbers were observed, probably reflecting loci where the whole repeat tract had been deleted, along with a few neighbouring bases. Such alleles were rounded to zero.

Mean expected span was calculated for each repeat number by averaging across all alleles of that size, across all loci and all possible genotypes, using allele frequencies derived from the full genome screen. We also explored the possibility of bias due to mutation ascertainment: when mutations create other parental alleles they sometimes fail to be revealed as deviations from Mendelian segregation. Generally, mutations are more likely to be detected in homozygotes, where alleles cannot mutate to their homologue's state, and in extreme alleles, where a preponderance of one-step mutations [18,19,35] makes mutation to the other parental allele less likely. As an alternative measure of expected span we therefore simulated 1000 randomised genotype families per allele and recorded the mean span both in cases where a mutation would have been detected, and over all possible mutations. Approximately 20–25% of mutations go undetected in a strict single-step model, and detectable mutations on average occur in genotypes with slightly reduced span. In practice, the effect is small (span reduced by 0.1 – 0.2 repeat units) and we chose to be conservative by using unadjusted expected span.

### Population genetic analysis

We analysed the published dataset of 783 autosomal microsatellites genotyped in the1048 samples of the human diversity cell line panel, drawn from 53 worldwide populations [29]. All statistical analyses were conducted using 'R' . General linear and linear models were constructed as full models including all second order interactions, and terms removed progressively to achieve the minimum AIC value using the function 'Step'. A sample of models were examined for the normality of their residuals and the impact of outliers. Small deviations from normality were common, as were outliers with medium leverage, but we found no evidence that one or a few unusual populations consistently drove the trends. For consistency, all population size estimates were taken from the Joshua project  and no attempt was made to correct for population sub-structure: if samples were described as being collected from country X we use the total population size of country X, while if the samples are described as being collected from region Y in country X we used the population size of region Y (see Additional 3).

## Authors' contributions

WA designed and conducted the analyses and drafted the paper. XX contributed microsatellite mutation data. JF contributed data to earlier drafts and helped write the paper.

## Supplementary Material

Additional file 1 **Coefficients for the predictor variables retained after backward simplification of general linear models (GLMs) fitted individually to data from 783 human microsatellite loci.** Click here for file

Additional file 2 **Coefficients for the predictor variables retained after backward simplification of general linear models (GLMs) fitted individually to data from 783 human microsatellite loci (with phylogenetic correction using CAIC).** Click here for file

Additional file 3 **Estimates of modern population size used in the current study** Click here for file
