# Supplementary material for: Heterozygosity increases microsatellite mutation rate, linking it to demographic history
Source: BMC Genet. 2008 Nov 14;9:72. doi: 10.1186/1471-2156-9-72 (PMC2615044; doi:10.1186/1471-2156-9-72)
Supplement: Additional file 2 — Coefficients for the predictor variables retained after backward simplification of general linear models (GLMs) fitted individually to data from 783 human microsatellite loci (with phylogenetic correction using CAIC). [file 1471-2156-9-72-S2.doc]

**Additional File 2**

Coefficients for the predictor variables retained after backward simplification of general linear models (GLMs) fitted individually to data from 783 microsatellite loci. Here we controlled for phylogenetic non-independence by applying the method of Comparative Analysis with Independent Contrasts (CAIC). For each variable, independent contrasts were extracted following the method of Purvis (see text). GLMs were then fitted to these contrasts. The response variable in all cases is mean length in each of 53 worldwide populations (represented by 52 independent contrasts). Icept is the estimate of the intercept. Predictor variables are: allele length distribution skew (Sk), log modern population size (Sz), distance from Africa in tens of thousands of kilometres (Ds) and distance from Africa squared (D2), plus all second order interactions. Blank cells indicate terms not retained in the minimum adequate model. All models were fitted in R and simplified using the STEP function to find the minimum AIC value.

| **Locus** | **Icept** | **Sk** | **Sz** | **Ds** | **D2** | **Sk:Sz** | **Sk:Ds** | **Sk:D2** | **Sz:Ds** | **Sz:D2** | **Ds:D2** |
| --- | --- | --- | --- | --- | --- | --- | --- | --- | --- | --- | --- |
| D12S1638 | 0.06 | 0.38 | 0.08 | 0.51 | -0.02 | -0.06 | -1.12 | 0.46 |  |  |  |
| D14S1007 | 0.02 | 1.44 |  | 1.00 | -0.36 |  | -3.90 | 3.32 |  |  |  |
| D9S1779 | 0.26 | 1.18 |  | 1.80 | -3.14 |  | -0.77 |  |  |  | 1.89 |
| D9S1825 | 0.95 | -0.15 | -0.19 | -6.43 | 13.34 |  | 3.08 | -3.58 | 1.83 | -1.63 | -5.31 |
| D7S2477 | 0.83 | -0.03 | -0.27 | 1.60 |  | 0.64 |  |  |  |  |  |
| D17S784 | 0.36 |  |  |  |  |  |  |  |  |  |  |
| D16S403 | 0.34 | 0.04 | -0.12 | 0.94 | -0.44 | 0.07 | 0.50 |  |  |  |  |
| D3S1262 | 0.25 | 0.53 |  |  |  |  |  |  |  |  |  |
| D10S189 | 0.25 | 0.30 | 0.03 | -0.99 | 4.55 |  | -1.14 | 1.17 |  | -0.23 | -2.97 |
| D20S103 | -0.01 | 0.11 | 0.14 | 3.40 | -4.84 |  | -1.25 | 1.84 | -1.06 | 1.09 |  |
| D8S261 | 0.40 | -0.16 | -0.06 | -0.14 | 4.49 | 0.28 |  |  | -0.42 |  | -2.65 |
| D8S262 | 0.04 | 0.18 | 0.08 | 0.17 | 2.95 |  | 0.74 |  | -0.64 |  | -2.01 |
| D8S560 | 1.24 | 0.06 | -0.32 | 3.71 | -7.38 |  | -2.53 | 4.55 | 0.72 |  |  |
| D4S403 | 0.41 | 0.00 |  | -0.32 | -2.38 |  | 2.39 | -1.55 |  |  | 1.79 |
| D5S408 | 0.39 | -0.11 |  | 1.93 | -3.66 |  |  |  |  |  | 1.66 |
| D4S408 | 0.50 | 0.02 | -0.07 | -3.78 | 7.84 |  | 2.57 | -3.83 | 0.83 | -1.06 | -1.87 |
| D16S3401 | 0.84 | 0.47 | -0.05 | 1.91 | 6.97 |  | -7.50 | 7.15 |  | -0.55 | -4.90 |
| D18S1390 | 0.40 |  |  |  |  |  |  |  |  |  |  |
| D8S503 | 0.42 | -0.05 |  |  |  |  |  |  |  |  |  |
| D10S212 | 0.26 |  |  |  |  |  |  |  |  |  |  |
| D1S235 | 0.75 |  |  |  |  |  |  |  |  |  |  |
| D11S969 | 0.33 | 0.03 | 0.06 | -1.90 | 5.48 |  | 0.26 |  |  | -0.12 | -3.07 |
| D3S1560 | 0.10 | 0.36 | 0.09 |  |  |  |  |  |  |  |  |
| D20S851 | 0.79 | -0.12 | -0.16 | -0.16 | -1.79 | 0.23 |  | 2.24 | -0.28 |  |  |
| D6S305 | 0.24 | 0.33 | -0.05 | 2.07 | -1.62 | 0.27 |  | 0.90 |  | -0.34 |  |
| D15S165 | 0.55 | 1.23 | -0.20 |  |  | 0.18 |  |  |  |  |  |
| D16S422 | 0.33 | 0.55 | 0.12 | 2.52 | -0.73 |  |  | 1.35 | -0.50 |  |  |
| D3S1311 | 0.42 | 0.48 |  |  |  |  |  |  |  |  |  |
| D1S2682 | 0.19 | 1.95 | -0.07 |  | 0.57 |  |  | -0.22 |  |  |  |
| D15S128 | 0.23 | 0.23 |  | 1.65 | -2.30 |  |  | 0.67 |  |  |  |
| D1S468 | 0.13 | 1.20 | -0.10 | 2.62 | -3.56 | 0.26 | -4.17 | 3.76 | -0.70 | 0.93 | 0.94 |
| D3S3630 | 0.33 | 0.47 |  | -1.02 | 2.47 |  |  |  |  |  | -1.18 |
| D2S2986 | 0.25 | 0.14 | 0.25 | 0.19 | 1.29 | -0.10 | 1.68 | -1.69 | -1.16 | 0.76 |  |
| D9S1838 | 0.31 | 0.38 |  |  | 0.41 |  |  |  |  |  |  |
| D13S285 | 0.19 | 0.63 |  | 1.70 |  |  | -1.28 |  |  |  |  |
| D3S3644 | 0.11 | 0.35 | 0.05 |  |  |  |  |  |  |  |  |
| D22S1169 | 0.26 | 0.40 | 0.08 | -0.47 |  |  | 0.50 |  |  |  |  |
| D9S1871 | 0.33 | 1.75 | -0.16 | 3.54 | -1.39 | 0.21 | -11.26 | 12.75 |  |  | -2.77 |
| D16S516 | 0.44 |  | -0.08 | -0.27 |  |  |  |  | 0.16 |  |  |
| D4S3360 | 0.11 | 0.55 | 0.06 | -1.22 | 1.97 | -0.10 | 1.38 |  | 0.61 | -0.58 | -0.87 |
| D6S2522 | 0.14 | 0.22 | -0.03 | -0.51 | 0.58 | -0.03 | 0.27 |  | 0.35 | -0.41 |  |
| D18S843 | -0.16 | 0.78 | 0.32 | 1.06 | 1.54 | -0.35 | -3.54 | 4.51 |  | -0.23 | -1.81 |
| NA-D10S-2 | 0.06 | 0.40 |  | 0.79 |  |  |  |  |  |  |  |
| D3S2409 | 0.26 | 0.37 | 0.05 |  |  | -0.13 |  |  |  |  |  |
| D6S1021 | 0.04 | 0.80 | 0.12 |  |  | -0.19 |  |  |  |  |  |
| D9S910 | 0.18 | 1.41 |  |  | -0.49 |  |  |  |  |  |  |
| D14S592 | 0.15 | 0.44 | 0.13 | -0.25 | 6.70 |  |  | -1.72 | -0.61 |  | -3.44 |
| D11S1993 | 0.05 | 1.35 | 0.13 | 0.55 | -0.19 |  |  | -2.60 |  |  | 2.13 |
| D5S2488 | 0.30 | 0.13 |  |  | -0.12 |  |  |  |  |  |  |
| D10S1221 | -0.06 | 1.26 | 0.13 | 2.53 | -4.49 |  |  |  | -0.70 | 0.58 | 2.01 |
| D10S1222 | 0.79 | 0.52 | -0.13 | -2.40 | 2.51 |  | 3.25 | -3.45 | 0.33 |  |  |
| D3S2418 | 0.12 | 0.90 | 0.11 | 2.10 | -1.55 | -0.14 |  |  |  |  |  |
| D6S1027 | 0.40 | 1.48 |  | -1.20 | 1.32 |  | -1.42 |  |  |  |  |
| D5S1480 | 0.41 | 1.48 |  | 3.04 | -4.03 |  | -11.80 | 13.06 |  |  |  |
| D18S858 | 0.00 | 0.97 | 0.15 | 1.30 | -3.65 | -0.38 |  |  | 0.19 |  | 1.61 |
| D15S652 | 0.45 | 0.13 | 0.11 | 0.82 | -0.70 |  |  |  | -0.76 | 0.56 |  |
| D10S1225 | 0.13 | 0.34 | 0.02 | 0.28 |  | -0.09 |  |  |  |  |  |
| D1S1627 | 0.17 | 0.32 | 0.00 | -0.28 | 3.36 |  | -1.10 | 0.46 |  | -0.28 | -1.63 |
| D12S2070 | 0.41 | -0.08 | -0.10 | -0.54 | 0.30 | 0.13 |  | -1.43 | 0.38 | -0.46 | 5.15 |
| D4S2394 | 0.33 | 0.06 | -0.08 | -0.02 | 2.07 | 0.29 |  |  |  |  | -1.41 |
| D13S779 | 0.30 | -0.01 | -0.06 | -0.62 | 2.08 |  |  | -0.22 | 0.13 |  | -1.08 |
| D12S1042 | -0.07 | 1.01 | 0.20 | 0.81 | 0.26 |  |  | -1.24 | -1.56 | 1.29 |  |
| D4S2397 | 0.29 | 0.28 |  |  | -0.19 |  |  | 0.46 |  |  |  |
| D2S1352 | 0.16 | 0.21 |  | 1.25 | -2.97 |  |  | -0.42 |  |  | 1.92 |
| D21S1440 | 0.29 |  |  |  |  |  |  |  |  |  |  |
| D2S1353 | 0.41 | -0.28 | 0.17 | 0.28 | -0.79 |  | 1.42 |  | -0.99 | 0.60 |  |
| D6S1031 | 0.43 | 0.75 |  |  |  |  |  |  |  |  |  |
| D15S655 | 0.00 | 0.60 | 0.01 | 0.46 | -0.44 | 0.06 | -0.87 | 0.77 |  |  |  |
| D12S1045 | 0.42 | 0.35 | 0.10 | -1.28 | 1.21 | -0.09 |  | -0.72 |  |  |  |
| D10S1230 | 0.15 | 0.24 | 0.05 | 1.06 | -2.48 |  |  |  |  |  | 1.33 |
| D1S3462 | 0.29 |  |  | -0.45 | 0.48 |  |  |  |  |  |  |
| D14S599 | 0.14 | 0.02 | 0.04 | 2.41 | -4.51 |  |  | 0.30 | -0.61 | 0.73 | 1.67 |
| D4S2361 | 0.27 | -0.02 | -0.10 | 1.07 | -2.23 | 0.11 |  |  |  |  | 0.99 |
| D10S1412 | 0.31 |  |  | -0.79 | 1.70 |  |  |  |  |  | -0.82 |
| D11S2362 | 0.27 |  |  | 0.88 | -0.57 |  |  |  |  |  |  |
| NA-D11S-1 | 0.38 |  |  |  |  |  |  |  |  |  |  |
| D3S4523 | 0.17 | 0.46 | 0.15 | 0.64 |  | -0.16 |  |  |  |  |  |
| D22S1045 | 0.13 | 0.58 |  |  | 0.32 |  |  |  |  |  |  |
| D16S748 | 0.31 | 0.37 | -0.12 | 0.84 | -0.34 | 0.22 | -2.14 | 0.93 |  |  |  |
| D16S2616 | 0.21 | -0.07 |  | 0.45 | -0.29 |  |  |  |  |  |  |
| NA-D1S-4 | 0.39 | 0.15 |  |  | 0.26 |  |  |  |  |  |  |
| D17S2193 | 0.06 | 0.52 | 0.10 | 0.51 | 2.05 | -0.05 | -1.85 | 1.41 | -0.23 |  | -1.16 |
| D18S1370 | -0.10 | 1.08 | 0.19 | 1.03 | -0.30 |  | -1.12 |  | -0.89 | 0.63 |  |
| D1S3720 | 0.30 |  |  |  |  |  |  |  |  |  |  |
| D1S1589 | 0.02 | 0.69 | 0.16 | 0.43 |  | -0.26 |  |  |  |  |  |
| D2S1356 | 0.58 | 0.32 | -0.14 | -1.26 | 0.94 | -0.15 |  |  | 1.14 | -0.82 |  |
| NA-D5S-1 | 0.42 | 0.07 | -0.08 | 1.69 | -3.78 | -0.07 | 2.23 | -2.00 |  | 0.25 | 2.10 |
| D16S3396 | 0.31 | 0.52 |  |  |  |  |  |  |  |  |  |
| D17S2195 | 0.30 | -0.05 | -0.07 | 1.15 | -1.84 |  |  | 1.18 |  |  |  |
| D9S2157 | 0.59 |  |  |  |  |  |  |  |  |  |  |
| D10S1208 | 0.07 | 0.95 | 0.07 |  |  |  |  |  |  |  |  |
| NA-D13S-1 | 0.55 | 0.00 | -0.12 | 0.21 | -0.76 |  | 0.21 |  |  | 0.17 |  |
| NA-D17S-1 | 0.23 |  | 0.08 | 0.15 | 0.00 |  |  |  | -0.49 | 0.43 |  |
| NA-D1S-1 | 0.11 | 0.59 | 0.06 | 0.90 | -0.90 |  |  |  | -0.53 | 0.52 |  |
| D18S1357 | 0.25 | 0.54 | -0.02 |  | -0.10 |  |  |  |  | -0.10 |  |
| NA-D18S-2 | 0.31 | 0.49 |  |  |  |  |  |  |  |  |  |
| D11S4459 | 0.05 | 0.21 | 0.05 | 0.84 | -1.48 |  |  |  | -0.27 | 0.24 | 0.67 |
| D6S1006 | 0.09 | 0.11 | 0.07 | 0.22 | -0.32 |  |  |  | -0.44 | 0.38 |  |
| D17S2180 | 0.25 |  |  |  |  |  |  |  |  |  |  |
| NA-D8S-2 | 0.05 | 0.97 | 0.05 | 0.33 | -0.56 | -0.16 | 0.49 |  |  |  |  |
| D17S1298 | 0.28 | 0.12 |  |  | -0.44 |  |  | 0.40 |  |  |  |
| NA-D7S-1 | -0.02 | 0.83 |  | 3.89 | -5.67 |  | -5.17 | 5.46 |  |  | 1.82 |
| D4S1625 | 0.16 |  | 0.23 | -0.71 | 3.23 |  |  |  |  | -0.36 | -1.57 |
| D1S1728 | 0.18 | 0.05 | 0.05 | 0.26 | 1.11 | 0.07 |  |  | -0.53 | 0.39 | -0.91 |
| D4S3243 | 0.24 | 0.21 | 0.00 | 0.48 | -1.53 |  |  | -2.48 |  | 0.17 | 1.83 |
| D10S2470 | 0.27 | 0.46 | 0.13 | -1.18 | 1.97 | -0.11 | -0.63 |  |  |  |  |
| D2S2952 | 0.85 | 0.15 | -0.10 | -1.80 | 0.46 |  | 1.67 |  |  | 0.40 |  |
| D11S4463 | 0.36 |  |  |  |  |  |  |  |  |  |  |
| D7S3046 | 0.21 | 0.50 | 0.30 | 1.98 | -1.21 |  |  |  | -1.65 | 1.07 |  |
| D7S3047 | 0.24 | -0.03 | 0.08 | -0.52 | -0.86 |  | 1.98 | -2.64 | -0.41 | 0.32 | 2.43 |
| D18S542 | 0.36 | 0.16 | -0.08 | -1.28 | 3.99 |  |  |  |  |  | -2.05 |
| D5S1456 | 0.60 |  | -0.07 |  |  |  |  |  |  |  |  |
| D22S683 | -0.21 | 2.40 | 0.28 |  | -0.47 | -0.54 |  |  |  |  |  |
| D16S539 | 0.31 | 0.44 | -0.10 | 0.21 | 0.46 |  | -0.93 |  | 0.69 | -0.62 |  |
| D13S1807 | 0.14 | -0.25 | 0.08 | 2.03 | -1.37 | 0.23 |  |  | -0.98 | 0.86 |  |
| D21S1432 | 1.21 | -1.47 | -0.28 | -4.30 | 10.57 | 0.59 | 1.24 |  |  |  | -5.29 |
| D6S2410 | 0.14 | 0.21 |  | 0.53 |  |  | -0.60 |  |  |  |  |
| D4S1644 | 0.12 | 0.04 | 0.08 | -0.03 | 0.02 |  | 0.65 |  |  | -0.11 |  |
| D2S1360 | 0.36 | 0.19 |  | 1.94 | -4.16 |  |  |  |  |  | 1.80 |
| NA-D10S-1 | 0.39 | 0.14 |  | -1.38 | 2.68 |  |  |  |  |  | -1.03 |
| NA-D1S-2 | 0.04 | 0.27 | -0.02 | 0.60 | 1.09 | 0.15 | -1.03 |  |  | -0.28 |  |
| NA-D1S-5 | 0.26 | 0.02 |  | -0.21 | 1.88 |  |  | -0.23 |  |  | -0.97 |
| D3S4529 | 0.19 | 0.33 | 0.03 |  | 0.56 |  |  |  |  | -0.13 |  |
| D20S1143 | 0.48 | -0.14 |  | -1.27 | 0.90 |  | 1.41 | -1.15 |  |  |  |
| D21S2052 | 0.39 | 0.09 | 0.09 | 0.00 | 0.14 |  | 0.78 |  |  | -0.18 |  |
| D1S3721 | 0.45 |  | 0.23 | 2.76 | -1.24 |  |  |  | -0.54 |  |  |
| D1S534 | 0.32 | 0.31 |  |  |  |  |  |  |  |  |  |
| D11S2363 | 0.52 | 0.29 | 0.21 | -0.20 | 1.30 |  |  |  | -0.39 |  |  |
| D2S427 | -0.39 | 1.43 | 0.32 | 3.82 | -7.69 | -0.51 |  |  |  |  | 3.57 |
| D18S535 | 0.68 | -0.13 | -0.20 | -5.58 | 6.79 |  | 10.24 | -11.04 | 1.17 | -0.97 |  |
| NA-D1S-3 | 0.22 | 0.29 | 0.02 | -1.02 | 2.46 |  |  | -1.16 | 0.59 | -0.82 |  |
| D5S2845 | 0.43 |  |  | 0.54 |  |  |  |  |  |  |  |
| D14S1426 | 0.09 | 1.21 | 0.32 | 1.33 | 0.65 | -0.15 | -2.54 |  | -1.28 | 0.96 |  |
| D7S3051 | 0.20 | 0.29 | 0.09 | 1.79 | -0.81 |  |  |  | -0.27 |  |  |
| NA-D16S-1 | 0.33 |  |  | 0.77 | -0.52 |  |  |  |  |  |  |
| D7S817 | 0.09 | 0.57 | 0.15 |  | 0.52 | -0.22 |  |  |  |  |  |
| D5S2849 | 0.25 |  | 0.08 |  |  |  |  |  |  |  |  |
| D8S2324 | 0.23 | 0.25 |  | 0.94 | -0.70 |  |  |  |  |  |  |
| NA-D8S-1 | 0.08 | 0.89 | 0.10 | -0.07 | 1.81 |  | -1.60 |  |  | -0.38 |  |
| D15S1507 | -0.15 | 0.90 | 0.09 | 0.25 | 0.45 | -0.18 |  | -0.84 | 0.18 |  |  |
| D6S2439 | 0.16 | 0.39 | 0.24 | -0.53 | 8.88 |  |  | -1.33 | -1.31 | 0.74 | -5.23 |
| D3S4545 | 0.62 |  |  | 2.09 | -1.12 |  |  |  |  |  |  |
| D6S2436 | 0.23 | 0.61 | 0.11 | 0.34 | 0.11 | 0.06 | -1.07 | 0.47 | -0.66 | 0.62 |  |
| D14S1434 | 0.17 | 0.45 |  |  | 0.21 |  |  |  |  |  |  |
| D2S2972 | 0.43 |  |  |  |  |  |  |  |  |  |  |
| D18S1371 | 0.46 | 0.02 | -0.01 | -1.65 | 1.66 | -0.09 |  |  | 0.83 | -0.66 |  |
| NA-D18S-1 | 0.24 | 0.26 | 0.10 | -1.20 | 4.34 |  |  |  | -0.32 |  | -2.23 |
| D2S2968 | 0.40 | -0.10 |  | -0.43 | 0.31 |  |  |  |  |  |  |
| NA-D6S-1 | 0.40 | 1.32 | 0.02 | 1.04 | -4.16 | -0.21 |  |  |  |  | 2.43 |
| D18S1376 | 0.19 | 0.07 | -0.04 |  |  |  |  |  |  |  |  |
| D17S2196 | 0.30 | 0.65 | -0.07 | -0.36 | 3.91 | 0.17 |  |  |  | -0.21 | -2.14 |
| NA-D9S-1 | 0.37 |  |  |  |  |  |  |  |  |  |  |
| D21S2055 | 0.88 | 4.92 | -0.15 | -1.24 | 5.68 | -1.20 | -6.72 |  | 1.17 |  |  |
| D7S3070 | 0.52 |  |  |  |  |  |  |  |  |  |  |
| NA-D14S-1 | -0.06 | 0.81 | 0.35 |  | 1.42 |  |  |  |  | -0.60 |  |
| D15S1515 | 0.11 | 0.51 |  | 0.90 | -0.56 |  |  |  |  |  |  |
| NA-D22S-1 | 0.88 | -0.32 | -0.16 | -1.49 | 1.88 | 0.56 |  | -1.52 |  |  |  |
| D8S1128 | 0.23 | 0.88 |  | 0.78 |  |  |  |  |  |  |  |
| D5S1457 | 0.06 | 1.27 | 0.16 | 0.88 | -3.01 | -0.40 |  |  | 0.35 |  | 1.97 |
| D22S689 | 0.56 | -0.19 |  |  |  |  |  |  |  |  |  |
| D9S922 | 0.34 | 0.35 | 0.02 |  |  | -0.13 |  |  |  |  |  |
| D19S1034 | 0.45 | -0.19 | -0.10 | -2.17 | 1.55 |  | 2.67 | -3.54 | 0.81 | -0.67 | 1.21 |
| D1S1594 | 0.21 | 0.07 |  | 0.47 |  |  | -0.33 |  |  |  |  |
| D16S3253 | -0.05 | 1.08 | 0.15 | 0.85 | -0.35 | -0.27 | -2.76 | 2.16 |  |  |  |
| D3S2427 | 0.41 |  |  |  |  |  |  |  |  |  |  |
| D4S2366 | 0.42 | 0.76 | 0.01 | -0.48 | 0.94 |  | -4.15 | 4.23 |  | -0.23 |  |
| D3S2387 | 0.49 | 0.15 | 0.34 | 0.43 |  | -0.33 | 1.15 |  | -0.49 |  |  |
| D19S586 | 0.48 | -0.10 | -0.19 |  | 0.39 | 0.23 |  | -0.73 |  |  |  |
| D13S787 | 0.46 | 0.02 | -0.14 | -1.86 | 4.80 | 0.22 |  |  |  |  | -2.33 |
| D2S1363 | 0.61 | 0.28 | -0.15 |  | -0.40 | 0.16 |  |  |  |  |  |
| D11S1998 | 0.18 | 0.94 | -0.02 | -1.20 | 7.18 | -0.15 | -3.08 | 2.93 | 0.67 |  | -6.52 |
| D7S1799 | 0.04 | 0.44 | -0.03 | 2.21 | -4.40 |  | -1.07 |  | 0.16 |  | 2.52 |
| D11S1999 | 0.51 | 0.35 | -0.10 | -1.69 | 2.95 |  | 1.06 |  |  | 0.27 | -1.80 |
| D6S1040 | 0.20 | 0.45 | -0.02 | 1.12 | -0.76 |  | -2.90 | 1.88 | 0.27 |  |  |
| D7S1818 | 0.31 |  |  | 1.05 | -2.19 |  |  |  |  |  | 1.26 |
| D7S3056 | 0.39 | -0.23 | -0.06 | -0.21 | -0.75 |  | 1.94 | -2.66 |  | 0.30 | 1.08 |
| D4S2367 | 0.16 | 0.37 | -0.01 | -0.49 | 0.48 |  |  |  | 0.50 | -0.42 |  |
| D17S1299 | 0.27 |  | 0.01 | 0.44 |  |  |  |  | -0.15 |  |  |
| D12S1052 | 0.39 |  | -0.01 | -0.98 | 1.00 |  |  |  |  | 0.17 |  |
| D8S1132 | 0.36 | 1.25 | -0.20 | -1.76 | 5.93 |  |  | -5.02 | 1.17 | -1.27 | -1.44 |
| D1S1596 | 0.25 | 0.53 | 0.03 | -1.10 | 0.22 | -0.33 | 1.59 |  |  | 0.19 |  |
| D15S642 | 0.39 | -0.05 |  |  | -0.50 |  |  | 0.59 |  |  |  |
| D9S925 | 0.19 | 0.68 | -0.08 | 0.68 | 0.26 | -0.15 | -1.13 |  | 0.74 | -0.67 |  |
| D2S1328 | 0.33 | 0.62 | -0.17 | 0.46 |  | 0.19 | -0.89 |  |  |  |  |
| D3S2432 | 0.64 | -0.01 | -0.29 | -2.17 | 9.44 | 0.30 | -3.86 | 11.13 | 2.01 | -3.37 | -14.88 |
| D1S1597 | 0.22 | 0.72 |  | 0.26 | 2.23 |  | -2.30 |  |  |  | -1.14 |
| D4S2368 | 0.07 | 0.63 | -0.06 | 1.16 | -0.33 |  | -2.29 | 1.65 |  |  |  |
| D11S2000 | 0.74 | 0.20 |  | 1.71 | -2.67 |  | -4.19 | 5.85 |  |  |  |
| D17S1301 | 0.17 | 0.12 | 0.00 | -0.34 | 0.48 |  |  |  | 0.40 | -0.33 |  |
| D4S3248 | 0.33 | -0.02 | -0.08 | -0.35 | 0.25 |  |  | 1.04 | 0.78 | -0.65 |  |
| D6S1959 | 0.08 | 0.14 | 0.04 | -0.17 | 2.98 | 0.08 | -0.46 |  |  | -0.24 | -1.44 |
| D1S3669 | 0.46 | 0.10 | 0.08 | -1.05 | 3.63 |  |  | 0.72 | -0.30 |  | -1.60 |
| D19S589 | 0.35 | 0.00 | -0.07 |  | 0.26 | 0.11 |  | -0.56 |  |  |  |
| D20S477 | 0.29 | 0.77 |  |  |  |  |  |  |  |  |  |
| D4S1647 | 0.30 | 0.27 | 0.02 | -1.31 | 3.42 | 0.24 |  |  | -0.19 |  | -1.24 |
| D5S816 | 0.46 | 0.07 | 0.05 | -1.54 | 2.23 |  | 3.41 | -3.49 |  | -0.15 |  |
| D14S606 | 0.26 | 0.06 |  | -0.63 | 1.32 |  |  |  |  |  | -0.67 |
| D7S3058 | 0.33 | 0.84 | 0.09 | -1.58 | 6.43 |  |  | 0.98 |  | -0.40 | -3.95 |
| D2S2944 | 0.25 | 0.42 |  | 1.92 | -3.97 |  |  |  |  |  | 1.91 |
| D11S2002 | 0.51 | 0.32 |  | -0.98 | 0.69 |  |  |  |  |  |  |
| D6S474 | 0.18 | 0.75 |  | -1.01 | 2.16 |  |  |  |  |  | -1.03 |
| D7S2846 | 0.42 |  |  |  |  |  |  |  |  |  |  |
| D14S1280 | 0.29 | 0.27 | -0.03 | -0.86 | 0.47 |  |  |  | 0.60 | -0.41 |  |
| D6S1009 | 0.44 | 0.29 | 0.48 | -0.05 |  |  |  |  | -0.48 |  |  |
| D7S1824 | -0.04 | 1.50 | 0.12 | 2.57 | -3.93 | -0.40 | -1.55 |  |  |  | 1.63 |
| D12S2078 | 0.34 | 0.28 | 0.20 | -1.31 | 5.37 |  |  |  | -1.27 | 0.87 | -3.09 |
| D3S1744 | 0.44 | 0.45 |  | -0.86 | 0.83 |  | -0.58 |  |  |  |  |
| D5S817 | 0.31 |  | 0.07 | -1.39 | 4.32 |  |  |  | -0.21 |  | -2.18 |
| D7S820 | 0.30 | 0.77 |  |  |  |  |  |  |  |  |  |
| D3S1763 | 0.55 | 0.27 |  |  |  |  |  |  |  |  |  |
| D5S1462 | 0.57 | -0.12 | 0.08 | -2.70 | 6.21 | 0.24 |  |  |  | -0.28 | -2.75 |
| D8S1136 | 0.24 | -0.04 | 0.01 | 1.55 | -1.04 | 0.23 |  | -0.50 | -0.91 | 0.68 |  |
| D7S1802 | 0.32 |  |  |  |  |  |  |  |  |  |  |
| D20S478 | 0.92 |  |  | 1.99 | -1.26 |  |  |  |  |  |  |
| D16S764 | 0.10 | 0.07 | 0.12 | -0.03 | -0.07 |  |  | 0.60 | -0.50 | 0.28 |  |
| D4S2417 | 0.21 | 0.10 | 0.20 | -0.27 |  | -0.12 |  |  |  |  |  |
| D1S1653 | 0.28 |  | 0.05 |  |  |  |  |  |  |  |  |
| D7S1804 | 0.35 | 0.76 | 0.24 | 1.97 | -1.51 |  |  |  | -1.79 | 1.42 |  |
| D14S608 | 0.43 | 1.42 | 0.24 | -2.92 | 7.52 |  | -5.22 | 6.91 |  | -0.39 | -4.23 |
| D13S793 | 0.20 | 0.51 | 0.08 | 1.92 | -2.63 |  |  | 1.36 | -0.76 | 0.80 |  |
| D19S591 | 0.48 |  | -0.07 | -0.20 |  |  |  |  | 0.26 |  |  |
| D20S480 | 0.56 | -0.31 | -0.11 | -0.59 | 0.36 | 0.32 |  |  |  |  |  |
| D11S2006 | 0.06 | -0.02 | 0.14 | 0.71 | -0.46 |  | 0.57 |  | -0.59 | 0.32 |  |
| D20S481 | 0.66 | 0.58 | -0.14 | -3.73 | 7.15 | -0.38 | 4.51 | -6.34 | 1.14 |  | -3.11 |
| D1S1660 | 0.12 | 0.61 | 0.07 |  |  |  |  |  |  |  |  |
| D9S930 | 0.53 | -0.13 | 0.12 | -4.06 | 8.31 |  |  | -1.17 | 0.66 | -0.51 | -3.56 |
| D11S1981 | 0.49 |  | 0.21 | -2.68 | 5.30 |  |  |  |  | -0.31 | -2.16 |
| D17S1290 | 0.48 |  |  |  |  |  |  |  |  |  |  |
| NA-D12S-1 | 0.88 | -0.46 | -0.11 | 2.89 | -2.14 | 0.54 | -1.50 |  | -1.57 | 1.84 |  |
| D3S1764 | 0.52 |  | -0.14 | -1.03 | 0.85 |  |  |  | 1.17 | -0.86 |  |
| D2S1334 | 0.70 | -0.34 | -0.12 | -0.55 | 2.96 | 0.21 |  |  |  |  | -1.72 |
| D2S410 | 0.28 |  |  |  |  |  |  |  |  |  |  |
| D2S434 | 0.10 | 0.45 | 0.05 |  | -0.12 |  |  |  |  |  |  |
| D12S395 | 0.41 | 0.16 |  |  |  |  |  |  |  |  |  |
| D12S372 | 0.14 | 0.21 |  | -0.23 | 1.37 |  |  | -0.52 |  |  | -0.73 |
| D1S549 | 0.04 | 0.41 | 0.35 | 0.76 | -1.30 | -0.35 |  | 1.07 | -0.67 | 0.46 |  |
| NA-D15S-1 | 0.09 | 0.17 | 0.18 | 0.26 | 1.47 |  |  |  | -1.08 | 0.74 | -1.14 |
| D8S1108 | 0.51 | -0.24 | -0.07 | -0.67 | 0.35 | 0.13 |  |  |  |  |  |
| D1S1609 | 0.30 | -0.03 | 0.02 | 1.57 | -1.29 | 0.45 |  |  | -1.00 | 0.84 |  |
| D15S643 | 0.11 | 0.18 | 0.08 | 0.12 | 1.45 |  | -1.28 |  |  |  | -0.93 |
| D13S796 | 0.08 | 0.46 | 0.13 | 0.07 | 2.95 |  | -0.97 |  | -0.98 | 0.73 | -1.96 |
| D20S482 | 0.16 |  | 0.14 | -0.42 | 0.37 |  |  |  |  | -0.13 |  |
| D2S1384 | 0.37 | 0.39 |  | 0.22 | -0.56 |  | -1.13 | 0.98 |  |  |  |
| D5S1501 | 0.27 | 0.09 |  |  |  |  |  |  |  |  |  |
| D9S2169 | 0.28 |  |  | 1.32 | -0.84 |  |  |  |  |  |  |
| D15S659 | 0.08 | 0.84 | 0.18 | 1.17 | -0.70 |  |  |  | -0.95 | 0.66 |  |
| D12S1064 | 0.18 | 0.23 | 0.23 | 1.49 | 0.38 |  | -2.50 | 3.17 | -1.49 | 1.26 | -2.02 |
| D11S2365 | 0.15 | 0.10 | 0.10 | -0.75 | 4.10 |  |  | -1.60 | -0.41 |  | -1.95 |
| D10S1239 | 0.42 | -0.09 | -0.07 |  |  | 0.10 |  |  |  |  |  |
| D6S1053 | 0.28 | 0.21 | 0.16 |  |  | -0.28 |  |  |  |  |  |
| D11S4464 | 0.54 | 0.02 | -0.12 | -1.88 | 2.16 |  |  | -0.48 | 1.04 | -0.92 |  |
| D13S800 | 0.03 | 0.19 | 0.12 | -0.46 | 2.89 |  | -0.44 |  |  | -0.13 | -1.54 |
| D9S934 | 0.35 | -0.23 | 0.13 | -1.84 | 4.14 |  | 1.96 | -2.11 | -0.71 | 0.54 | -1.96 |
| D18S877 | 0.27 | 0.34 | -0.09 | -0.39 |  |  |  |  | 0.15 |  |  |
| D2S1391 | 0.18 | 0.17 |  |  |  |  |  |  |  |  |  |
| D19S714 | 0.49 | 0.24 | -0.01 | -1.08 | 0.66 |  |  |  | 0.89 | -0.66 |  |
| D5S2500 | 0.14 | 0.96 |  | -0.29 | 0.77 |  | -1.64 |  |  |  |  |
| D5S2501 | 0.39 | -0.11 | -0.03 | -1.01 | 0.56 |  | 1.53 | -0.88 | 0.14 |  |  |
| D3S2460 | 0.08 | 0.42 | 0.11 |  |  | -0.16 |  |  |  |  |  |
| D6S1056 | 0.60 |  |  | -1.88 | 4.21 |  |  |  |  |  | -2.07 |
| D2S1394 | 0.13 | 0.13 |  | 0.49 | -0.32 |  | -0.68 | 0.43 |  |  |  |
| D1S551 | 0.20 | -0.24 | -0.05 |  | 0.14 | 0.36 |  |  |  | -0.07 |  |
| D8S592 | 0.36 |  |  | -1.41 | 3.73 |  |  |  |  |  | -1.88 |
| D11S1392 | 0.18 |  | 0.15 | 0.74 | -0.54 |  |  |  | -0.68 | 0.50 |  |
| D12S373 | 0.22 |  | 0.04 | 0.74 | -0.58 |  |  |  | -0.58 | 0.49 |  |
| D18S851 | 0.27 |  |  |  | 0.15 |  |  |  |  |  |  |
| D5S820 | 0.42 |  | 0.00 |  | -0.08 |  |  |  |  | 0.12 |  |
| D3S1766 | 0.22 |  |  |  |  |  |  |  |  |  |  |
| D3S2398 | 0.14 | 0.48 | 0.12 |  |  | -0.36 |  |  |  |  |  |
| D21S1446 | 0.23 | 0.45 | -0.03 | -0.93 | 0.53 |  |  | 0.86 | 0.41 | -0.38 |  |
| NA-D4S-1 | -0.09 | 1.35 | 0.01 | 2.50 | -0.52 |  | -3.33 | 6.36 | -0.37 |  | -2.78 |
| D10S1423 | 0.06 | 0.53 | 0.12 | -0.08 | 2.07 | -0.18 | -1.63 | 0.91 |  |  | -1.31 |
| D10S1425 | 0.23 | 0.11 |  | 0.41 | -1.90 |  |  | 1.85 |  |  | 0.91 |
| D2S1776 | 0.21 | 0.19 |  | 0.58 | -1.52 |  |  |  |  |  | 0.72 |
| D9S1118 | -0.15 | 1.77 | 0.07 | 0.32 | -1.33 | -0.30 | 4.27 | -4.68 |  |  | 1.09 |
| D16S2621 | 0.68 | 0.51 | -0.19 |  | -0.37 | 0.21 |  |  |  |  |  |
| D16S769 | 0.14 | 0.25 |  |  |  |  |  |  |  |  |  |
| D4S2632 | 0.97 |  | -0.20 | -1.21 | 0.92 |  |  |  | 1.36 | -1.12 |  |
| D2S1780 | 0.23 | 0.04 |  | 1.99 | -4.01 |  |  | -1.08 |  |  | 2.44 |
| D1S2134 | 0.50 | 0.63 | 0.19 | -0.72 | -6.56 | -0.30 | 5.58 | -3.67 |  | 0.38 | 4.31 |
| D3S3038 | 0.33 | 0.11 |  |  |  |  |  |  |  |  |  |
| D7S2204 | 0.42 | 0.31 | -0.09 | 1.15 | -1.08 | 0.24 | 0.85 |  | -0.54 |  |  |
| D10S1426 | 0.22 | 0.03 |  |  | 0.25 |  |  | -0.43 |  |  |  |
| D15S816 | 0.22 |  |  |  |  |  |  |  |  |  |  |
| D12S1294 | 0.62 |  |  | -0.37 |  |  |  |  |  |  |  |
| D14S742 | 0.22 |  |  |  |  |  |  |  |  |  |  |
| D1S518 | 0.78 | -0.10 | -0.12 | -4.17 | 10.64 |  |  | -1.23 | 0.90 | -0.78 | -4.98 |
| D5S1470 | 0.32 | 0.00 |  | 1.03 | -0.52 |  |  | -1.08 |  |  |  |
| D4S1627 | 0.12 | 0.33 | 0.18 | 1.25 | -0.31 |  | -2.32 | 2.29 | -1.15 | 1.06 | -1.04 |
| D9S301 | 0.23 | 0.91 | 0.02 | -0.76 | 0.51 |  | -0.35 |  |  | 0.17 |  |
| D18S1364 | 0.42 | 0.54 |  | -1.04 | 0.63 |  |  |  |  |  |  |
| D3S3039 | 0.03 | 0.30 | 0.18 | -0.54 | 1.95 | -0.14 |  |  | -0.49 | 0.30 | -1.07 |
| D8S1179 | 0.52 | 0.33 | 0.12 | -2.41 | 5.39 |  | -2.15 | 2.77 |  | -0.25 | -3.02 |
| D13S317 | 0.08 | 0.94 | 0.11 |  |  | -0.20 |  |  |  |  |  |
| D6S1277 | 0.27 | -0.11 | 0.13 | 0.29 |  |  |  |  | -0.22 |  |  |
| D9S1120 | 0.43 | -0.08 | -0.40 |  | 0.67 | 0.52 |  | -1.09 |  |  |  |
| D16S2624 | 0.36 | 0.00 | -0.01 | -1.16 | 2.26 |  | 0.81 |  |  | 0.20 | -1.49 |
| D3S3045 | 0.40 | 0.53 | -0.13 |  | 0.29 | 0.29 |  | -0.92 |  |  |  |
| D10S1430 | 0.13 | 0.78 |  | -0.32 | 0.35 |  | -0.50 |  |  |  |  |
| D5S2505 | 0.33 | 0.08 | -0.09 | 0.92 | -0.80 | 0.53 | 3.16 | -3.21 | -1.54 | 1.37 |  |
| D12S1300 | 0.16 | 0.30 | 0.01 | -1.03 | 3.92 | 0.06 |  |  |  | -0.14 | -2.00 |
| D15S818 | 0.08 | 0.07 | -0.02 | 1.58 | -4.26 |  | 0.33 | -0.70 |  | 0.13 | 3.19 |
| D2S1788 | 0.26 | 1.63 | -0.07 | 2.42 | -3.34 | -0.22 | -3.13 | 2.72 |  | 0.35 |  |
| D13S894 | 0.10 |  | 0.09 | 0.31 | -0.10 |  |  |  |  | -0.10 |  |
| D9S1121 | 0.01 | 0.60 | 0.02 | 2.19 | -0.87 | 0.15 | -0.81 |  | -0.31 |  |  |
| D10S1432 | 0.44 | 0.01 | -0.16 | -1.90 | 3.21 |  | 0.55 |  | 1.06 | -0.93 | -1.23 |
| D10S1435 | 0.27 | -0.16 | 0.13 | -0.90 | 1.15 |  | 1.71 | -1.30 | -0.26 |  |  |
| D2S1790 | 0.48 | 0.13 | -0.08 | -0.95 | 2.78 |  |  | 0.23 |  |  | -1.96 |
| D15S822 | 0.91 | -0.09 | -0.13 | -3.86 | 4.51 | 0.32 | 5.77 | -8.68 |  | 0.19 |  |
| D9S1122 | 0.17 | 0.09 |  | 0.22 |  |  | -0.26 |  |  |  |  |
| D5S1725 | 0.28 | 0.51 | -0.10 | 0.79 | -1.72 |  |  |  |  |  | 1.01 |
| D4S1629 | -0.04 | 0.74 | -0.01 | 0.64 | -0.38 | -0.13 |  |  |  |  |  |
| D20S201 | 0.36 | 0.12 |  |  |  |  |  |  |  |  |  |
| D3S1768 | 0.10 | 0.19 | 0.11 | -0.46 | 1.58 | -0.06 |  | 0.40 |  |  | -1.22 |
| D17S974 | -0.01 | 0.44 | 0.08 | 0.52 | -0.26 |  | -2.22 | 1.78 |  |  |  |
| D3S1746 | 0.23 | 0.20 | 0.08 | 0.92 |  | 0.39 | -0.90 |  | -0.29 |  |  |
| D2S441 | 0.15 | 0.01 | 0.05 | -0.88 | 2.47 |  | 1.53 | -1.66 |  | -0.18 |  |
| D2S405 | 0.23 | 0.10 |  |  | 0.22 |  |  |  |  |  |  |
| D8S1110 | 0.58 |  | -0.16 | 2.29 | -5.17 |  |  |  |  | 0.35 | 2.29 |
| D11S2371 | 0.22 |  |  |  |  |  |  |  |  |  |  |
| D12S1301 | 0.21 | 0.35 | -0.02 |  | 0.42 |  |  |  |  | 0.09 |  |
| D14S587 | 0.12 | 1.17 | 0.12 | -1.09 | 4.26 | -0.62 |  | 1.83 |  |  | -3.56 |
| D22S686 | 0.05 | 1.07 |  |  |  |  |  |  |  |  |  |
| D11S1984 | 0.41 |  |  | 1.48 | -0.92 |  |  |  |  |  |  |
| D4S2431 | 0.12 | 0.17 | 0.14 | 0.59 | 0.24 |  | -0.49 |  | -0.60 | 0.36 |  |
| D8S1477 | 0.26 |  | -0.04 | 2.45 | -3.97 |  |  |  |  | 0.14 | 1.45 |
| D2S1399 | 0.32 | 0.87 | 0.08 | -1.16 | 8.03 | -0.41 |  | -1.59 |  | -0.39 | -4.20 |
| D2S1400 | -0.02 | 0.46 | 0.18 | 0.27 | -3.13 | -0.35 | 2.67 | -1.64 |  |  | 2.06 |
| D14S617 | 0.28 | 0.32 | -0.02 | -0.39 | 3.38 | 0.30 |  |  | -0.35 |  | -1.87 |
| D9S938 | 0.12 | 0.85 | -0.17 | 2.20 | -4.84 |  | -3.43 | 3.98 | 0.43 |  | 2.23 |
| D13S895 | 0.26 |  |  | 0.53 | -0.29 |  |  |  |  |  |  |
| D1S1677 | 0.21 | 0.12 | -0.11 | 0.63 | -0.23 | 0.06 | -0.99 | 0.57 |  |  |  |
| D10S1248 | 0.30 | 0.10 |  |  | -0.11 |  |  |  |  |  |  |
| D13S1493 | 0.32 | 0.00 | 0.02 | -1.31 | 2.63 |  | 2.12 | -1.00 |  | -0.19 | -1.17 |
| D19S433 | 0.22 |  |  | 1.34 | -2.72 |  |  |  |  |  | 1.23 |
| D10S677 | 0.31 | 0.09 |  |  | 0.34 |  |  |  |  |  |  |
| D1S1612 | 0.46 | -0.27 | -0.04 | -0.09 | -0.06 | 0.15 | 2.22 | -1.41 |  |  |  |
| D21S1437 | 0.48 | 0.22 | -0.12 | -0.85 | -0.26 |  |  | 1.68 | 0.99 | -0.73 |  |
| D7S1808 | 0.33 | -0.12 | -0.07 | 0.45 |  | 0.19 |  |  |  |  |  |
| D16S753 | 0.27 | 0.19 |  | 0.34 |  |  |  |  |  |  |  |
| D14S588 | 0.26 | 0.13 | 0.10 | -2.00 | 3.30 |  | 2.04 | -1.49 |  |  | -1.06 |
| D3S2403 | 0.13 | 0.48 | 0.25 | -0.54 | 3.58 | -0.10 | -0.61 |  | -0.87 | 0.66 | -2.07 |
| D1S1679 | 0.20 |  | 0.13 | 0.21 | 0.38 |  |  |  | -0.23 |  |  |
| D7S3061 | 0.46 | -0.03 | 0.10 |  | -0.87 |  |  | 0.75 |  |  |  |
| D11S1986 | 0.42 | 1.41 | -0.12 | -0.44 | 0.08 | 0.29 | 2.35 | -2.83 |  | 0.35 |  |
| D8S1113 | 0.55 | -0.33 | 0.00 | -1.16 | 2.53 |  | 2.57 | -2.26 | -0.20 |  |  |
| D17S1294 | 0.29 |  |  |  |  |  |  |  |  |  |  |
| D10S2327 | 0.19 | 0.71 | 0.01 | -1.12 | 5.49 |  | -0.98 |  |  | -0.25 | -2.72 |
| D6S1017 | 0.50 | -0.16 |  | -1.08 | 0.86 |  | 4.70 | -2.86 |  |  |  |
| D17S1308 | 0.23 | 0.04 |  |  |  |  |  |  |  |  |  |
| D5S211 | 0.16 | 0.20 |  |  |  |  |  |  |  |  |  |
| D19S246 | 0.12 | 1.44 |  | 0.83 | -0.56 |  |  |  |  |  |  |
| D19S245 | 0.28 | -0.16 |  |  |  |  |  |  |  |  |  |
| D19S254 | 0.48 | -0.05 | -0.02 | -0.92 | 3.41 |  |  | 2.19 |  | -0.22 | -2.59 |
| D12S269 | 0.13 | 0.33 | 0.01 | 0.08 | 1.00 |  | -0.72 |  |  | -0.12 |  |
| D7S559 | 0.07 | 0.23 | 0.12 |  |  | -0.16 |  |  |  |  |  |
| D22S345 | 0.13 | 0.10 |  |  | 0.12 |  |  |  |  |  |  |
| NA-D12S-2 | 0.48 |  | -0.03 |  | -0.29 |  |  |  |  | 0.20 |  |
| F13A1-D6S | 0.67 | -0.02 | 0.01 | 2.08 | 2.93 |  |  | -3.84 | -1.69 | 1.83 |  |
| TPO-D2S | 0.06 | 0.41 | 0.10 | -0.82 | 3.43 |  |  |  | -0.19 |  | -1.83 |
| D20S159 | 0.73 |  |  |  |  |  |  |  |  |  |  |
| D21S1411 | 0.42 | 0.10 | 0.10 | -0.83 | 0.85 | -0.22 |  |  |  | 0.21 |  |
| D20S164 | 0.26 | -0.15 | 0.00 | 0.16 | -2.20 |  | 1.23 |  | 0.54 | -0.57 | 1.80 |
| D11S1304 | 0.03 | 1.42 |  | 1.95 | -1.75 |  | -1.35 | 1.26 |  |  |  |
| D20S451 | 0.25 | 0.16 |  | -0.27 | -1.26 |  | 2.29 | -1.62 |  |  | 1.22 |
| D12S297 | 1.39 | 0.11 | -0.25 |  | 0.76 | 0.25 |  |  |  |  |  |
| D6S942 | 0.12 | 0.13 |  |  |  |  |  |  |  |  |  |
| D8S1048 | 0.17 | 0.04 | 0.06 | -0.70 | 0.89 |  | 0.59 | -0.55 | -0.08 |  |  |
| D22S532 | 0.11 | 0.05 | 0.07 | 0.05 | 2.35 |  |  | -0.46 | -0.42 | 0.42 | -1.47 |
| D8S373 | 0.29 |  |  | 1.03 | -2.02 |  |  |  |  |  | 1.20 |
| D19S559 | 0.27 | 0.02 | -0.02 | -0.54 | 2.52 |  |  | -0.45 |  | -0.12 | -0.92 |
| D4S1652 | 0.19 |  |  | 0.17 |  |  |  |  |  |  |  |
| D7S821 | 0.34 |  |  |  | 0.11 |  |  |  |  |  |  |
| D1S1665 | 0.26 |  |  |  |  |  |  |  |  |  |  |
| D6S1051 | 0.21 |  |  |  | 0.24 |  |  |  |  |  |  |
| D5S1505 | 0.03 | 0.26 | 0.16 | 2.36 | -2.36 |  | -1.53 | 1.28 | -0.81 | 0.78 |  |
| D4S2623 | 0.07 | 1.06 | 0.16 | 1.08 | 0.61 | -0.18 |  |  |  | -0.32 |  |
| GATA194H05Z | 0.12 | 0.52 |  |  |  |  |  |  |  |  |  |
| GTTTT002P | 0.61 | -0.32 | -0.13 | -1.73 | 7.31 | 0.16 | 1.22 | -1.14 | -0.52 |  | -3.47 |
| GGAA23C07 | 0.40 | -0.36 | -0.13 | -1.02 | 2.63 | 0.32 | 0.95 |  |  |  | -1.32 |
| CATC015 | 0.40 | -0.10 | -0.08 |  |  | 0.07 |  |  |  |  |  |
| MFD424-TTTA003 | 0.04 | 1.37 | 0.10 |  | -0.56 | -0.30 |  | 0.76 |  | 0.23 |  |
| GATA23G09 | 0.18 | 0.31 | -0.06 | -0.24 | 0.63 |  | -1.70 | 2.88 | 0.46 | -0.38 | -0.99 |
| AAT238 | 0.15 | 0.19 | 0.02 | -0.24 | 1.88 |  |  |  |  | -0.12 | -1.03 |
| TTTA063P | 0.39 |  |  | -0.38 | 4.11 |  |  |  |  |  | -2.06 |
| ATA008 | 0.21 | 0.11 | -0.07 | 0.14 |  | 0.10 |  |  |  |  |  |
| ATA43C09M | 0.06 | 0.72 | 0.02 | -1.67 | 5.35 |  |  | -0.60 |  | -0.20 | -2.41 |
| ATA20F08P | 0.16 | 0.36 | -0.01 | -0.51 | 0.46 |  |  | 0.33 |  | -0.07 |  |
| AGAT135 | 0.04 | 0.25 | 0.09 | 1.45 | -1.07 |  | -0.81 |  | -0.68 | 0.78 |  |
| AAT267 | 0.23 | -0.05 | -0.06 | -0.33 | 1.17 | 0.02 |  | 0.11 | 0.20 | -0.24 | -0.70 |
| AGAT017 | 0.15 | 0.13 | 0.05 |  |  |  |  |  |  |  |  |
| AAT258 | 0.28 |  | 0.00 | 0.79 | -2.59 |  |  |  |  | 0.27 | 1.39 |
| GATA2B02Z | 0.16 | 0.96 | 0.05 | 1.17 | -1.95 |  | 1.01 |  | -0.92 | 0.94 |  |
| AGAT143 | 0.25 |  |  |  |  |  |  |  |  |  |  |
| GATA152F05L | 0.21 |  |  | 0.19 |  |  |  |  |  |  |  |
| GATA193D02 | 0.21 |  |  | -0.26 | 1.62 |  |  |  |  |  | -0.78 |
| AAT259 | 0.18 |  |  |  |  |  |  |  |  |  |  |
| GATA113H03 | 0.42 |  | -0.02 | -1.08 | 3.07 |  |  |  | -0.18 |  | -1.46 |
| AAT252 | 0.11 | 0.43 |  | 0.62 | -0.90 |  |  | 0.42 |  |  |  |
| GGAA20F08 | 0.31 | -0.03 |  | 0.05 |  |  | 0.99 |  |  |  |  |
| GATA13C08M | 0.33 |  |  | -0.55 | 0.37 |  |  |  |  |  |  |
| AGAT119M | 0.18 | 0.60 | -0.06 | 0.09 | 0.11 |  | -2.26 | 2.83 | 0.52 | -0.60 |  |
| ATA73A08M | 0.24 |  | 0.24 | 0.14 | 0.01 |  |  |  | -0.73 | 0.44 |  |
| ATA38A05 | 0.11 | 0.70 |  |  | 0.60 |  |  | -0.81 |  |  |  |
| TATC028 | 0.05 | 1.12 | 0.04 |  | 0.99 | -0.14 |  | -1.01 |  | -0.20 |  |
| AGAT118 | 0.20 | 0.14 |  |  |  |  |  |  |  |  |  |
| AAT200 | 0.08 | 0.81 |  |  |  |  |  |  |  |  |  |
| GATA51H01 | 0.27 | 1.08 | -0.09 | 0.85 | -4.80 |  | 1.22 |  |  | 0.35 | 1.95 |
| GATA135F02P | 0.33 |  |  | -0.45 | 2.43 |  |  |  |  |  | -1.41 |
| AGAT141 | 0.27 |  | 0.08 |  |  |  |  |  |  |  |  |
| GATA88F03P | 0.40 | -0.30 | -0.12 | 1.18 | -0.79 | 0.45 |  |  |  |  |  |
| GAT004 | 0.09 | 0.27 |  | -0.09 |  |  | 0.27 |  |  |  |  |
| ATA2E04 | 0.16 | 0.07 | 0.31 | 0.02 | 2.17 |  |  | 0.62 | -1.80 | 1.45 | -2.11 |
| GATA64F05 | 0.09 | 0.35 | 0.07 | 1.50 | -0.97 |  |  |  | -0.78 | 0.51 |  |
| ATA009 | 0.06 | 0.81 | 0.25 | -0.28 | 0.10 | -0.38 | 0.79 |  |  | -0.12 |  |
| AAT243 | 0.30 |  |  |  |  |  |  |  |  |  |  |
| AATA011 | 0.16 | 0.07 |  | 0.64 | -0.45 |  |  |  |  |  |  |
| TTTA049 | 0.37 | -0.06 | -0.03 | -0.30 | -0.45 |  |  | 0.59 | 0.20 |  |  |
| GATA28H06 | 0.12 | 0.01 | 0.19 | -0.21 | -0.26 |  | 0.44 |  | -0.78 | 0.63 |  |
| GAAT1A5 | 0.28 |  | 0.05 |  | -0.14 |  |  |  |  |  |  |
| AAT055ZM | 0.28 | 0.39 |  | -0.87 | 0.94 |  | 2.47 | -2.29 |  |  |  |
| GATA056 | 0.17 |  | 0.05 |  |  |  |  |  |  |  |  |
| AAT263P | 0.06 | 0.63 | 0.32 | 1.15 | -1.80 | -0.20 | 1.16 |  | -1.55 | 1.31 |  |
| AGAT117 | 0.33 | 0.08 |  | -1.37 | 2.68 |  |  |  |  |  | -1.24 |
| GATA194B06P | 0.44 | 0.86 | 0.10 |  |  | -0.17 |  |  |  |  |  |
| ATA47C04P | -0.01 | 0.41 | 0.08 | 1.82 | -1.91 |  | -2.83 | 2.93 | -0.54 | 0.50 |  |
| GATA174G01 | 0.45 | -0.48 | -0.14 | 0.20 |  | 0.47 |  |  |  |  |  |
| GATA10H05 | 0.25 | 0.21 |  |  |  |  |  |  |  |  |  |
| GATA130A05M | 0.32 | -0.12 | 0.18 | -1.21 | 2.54 |  | 1.46 | -1.84 | -1.19 | 0.99 | -1.06 |
| GATA66D01 | 0.16 | 1.48 | 0.01 | 2.81 | -6.64 |  | -6.94 | 9.32 |  | 0.32 | 2.40 |
| GATA181G08M | 0.41 | 0.21 | 0.09 | -0.07 | -0.22 |  |  | 1.47 | -0.36 |  |  |
| GATA70F12M | 0.28 | 0.52 | -0.10 | 0.98 | -0.76 |  |  |  |  |  |  |
| ATA16D09 | 0.58 |  |  | 1.59 | -1.24 |  |  |  |  |  |  |
| GATA148G10P | 0.09 | 0.16 | 0.09 | 0.87 | -0.66 |  |  |  | -0.53 | 0.38 |  |
| AAAT105ZP | 0.19 |  | 0.05 |  |  |  |  |  |  |  |  |
| GATA139A04P | 0.37 |  |  |  |  |  |  |  |  |  |  |
| TAGA002M | 0.29 | 0.31 |  |  |  |  |  |  |  |  |  |
| GATA8H05 | 0.42 | -0.31 | 0.20 | -1.65 | 1.58 |  | 2.81 | -3.04 | -0.89 | 0.93 |  |
| AGAT093 | 0.14 | 0.03 | 0.07 | 0.08 | 1.71 |  |  | 0.43 |  | -0.16 | -1.16 |
| GATA63F01 | 0.24 | 0.20 |  | 1.01 | -2.48 |  |  |  |  |  | 1.20 |
| GATA43F06 | 0.86 |  |  |  |  |  |  |  |  |  |  |
| GATA126A06M | 0.12 | 0.23 | 0.01 |  |  | -0.06 |  |  |  |  |  |
| GATA91D12M | 0.21 | 0.23 | 0.03 | 0.05 | 2.56 |  | -1.30 |  | -0.60 | 0.44 | -1.56 |
| GATA194A05M | 0.26 |  | 0.09 | 0.40 |  |  |  |  | -0.19 |  |  |
| ATA29E07M | 0.45 | 0.45 |  |  |  |  |  |  |  |  |  |
| GATA149B10M | 0.17 | -0.03 | 0.00 | 0.13 |  | 0.05 |  |  |  |  |  |
| GATA29A06M | 0.09 | 0.35 | -0.07 | 0.97 | -1.87 | 0.15 |  |  |  |  | 0.81 |
| AAT203 | 0.34 | -0.09 | -0.17 | -0.38 | 0.57 | 0.14 |  | -0.46 | 0.46 | -0.37 |  |
| GATA23A02 | 0.24 | 0.00 | -0.01 | -0.57 | 0.72 |  |  | -0.52 | 0.48 | -0.41 |  |
| AGAT021 | 0.19 | 0.13 |  |  |  |  |  |  |  |  |  |
| MFD433-AGAT010 | 0.07 | 0.23 | 0.23 | 1.22 | -2.23 | -0.12 |  | 0.72 | -1.18 | 1.09 |  |
| TATC046 | 0.16 |  | 0.02 | 0.46 |  |  |  |  | -0.11 |  |  |
| GATA131D09 | 0.11 | -0.12 | 0.07 | 0.98 | -0.72 | 0.15 |  |  | -0.86 | 0.57 |  |
| ATCT053P | 0.25 | 0.37 |  |  |  |  |  |  |  |  |  |
| GATA178C11M | 0.12 |  | 0.02 | 0.48 | -0.31 |  |  |  | -0.07 |  |  |
| GATA146B10 | 0.31 | -0.08 | -0.01 | -1.04 | 2.15 | 0.09 |  |  |  |  | -1.04 |
| AAT264M | 0.24 | 0.27 | 0.04 | -0.95 | 2.12 |  |  |  |  | -0.11 | -0.88 |
| TAT024Z | 0.41 | 0.01 | -0.16 | -2.23 | 2.56 |  | 1.79 | -1.85 | 1.24 | -1.09 |  |
| GATA87B02 | 0.21 | -0.15 | 0.00 |  | 0.13 | 0.08 |  |  |  | -0.07 |  |
| AGAT128 | 0.27 | -0.15 |  |  |  |  |  |  |  |  |  |
| AAC023 | 0.13 | 0.20 | 0.07 | -0.65 | 1.44 |  | 1.14 | -1.02 | -0.41 | 0.41 | -0.73 |
| GGAT2G03 | 0.57 | 0.65 |  |  |  |  |  |  |  |  |  |
| GATA146D07 | 0.10 | 0.26 | 0.05 |  |  |  |  |  |  |  |  |
| ATC3D09 | 0.43 |  | 0.15 | 0.27 |  |  |  |  | -0.23 |  |  |
| GATA68D03 | 0.46 | -0.13 | -0.02 | -0.76 |  | 0.22 | 1.04 |  |  |  |  |
| ATC4D07 | 0.17 | 0.15 |  | -0.13 |  |  | 0.24 |  |  |  |  |
| TTTA033 | 0.07 | 0.91 | -0.04 | 0.50 | -0.36 |  | -1.25 | 1.14 |  |  |  |
| GATA152F04M | 0.65 | -0.50 | -0.29 | -0.13 | 0.28 | 0.44 |  |  | 0.73 | -0.58 |  |
| ATA85B10P | 0.42 | 0.05 | -0.07 | -1.43 | 0.95 |  |  | 0.83 | 0.88 | -0.82 |  |
| AAT071 | 0.33 | 0.17 |  | -0.67 | 0.45 |  |  |  |  |  |  |
| ATA21H07M | 0.03 | 1.15 | 0.03 |  | -0.13 | -0.23 |  |  |  | 0.10 |  |
| GATA92B06P | 0.17 | 0.53 | -0.11 | 0.41 | -0.27 |  | -2.76 | 2.51 | 0.69 | -0.56 |  |
| GATA72A06 | 0.48 | -0.18 | 0.14 | -1.67 | 2.27 |  | 2.01 | -2.10 | -0.37 |  |  |
| AAC030 | 0.18 | 0.26 | 0.00 | -0.24 | 0.85 | 0.17 | -0.57 |  |  | -0.23 |  |
| TTTA040 | 0.36 | -0.02 | 0.07 | -1.70 | 3.59 |  |  | -0.49 |  | -0.14 | -1.31 |
| ATA57D10M | 0.11 | 0.58 |  | 0.49 | -0.35 |  |  |  |  |  |  |
| GGAA22B10P | 0.67 |  |  |  |  |  |  |  |  |  |  |
| CTAT012 | 0.24 | 0.63 | -0.06 | 0.51 | 1.03 | 0.25 | -1.67 |  |  | -0.19 |  |
| AAAT028 | 0.30 | 0.04 |  | -1.05 | 0.47 |  | 0.90 | -0.51 |  |  |  |
| AAT246 | 0.02 | 0.57 | 0.29 | -0.58 | 2.77 | -0.13 |  |  | -0.93 | 0.57 | -1.59 |
| TTTAT002Z | 0.34 |  |  |  |  |  |  |  |  |  |  |
| ATT015 | 0.39 | 0.26 | -0.04 | 0.27 | -0.39 | 0.35 |  |  | -1.04 | 0.79 |  |
| TAAAA006 | 0.22 | 0.87 |  | 3.87 | -8.29 |  | -5.84 | 9.54 |  |  | 3.62 |
| ATCT018 | 0.31 | 0.09 | -0.09 | 0.19 |  | 0.29 |  |  |  |  |  |
| ATA21F01 | 0.11 | 0.04 | 0.07 | 0.49 | -0.28 |  | -1.02 | 0.74 |  |  |  |
| GATA29 | -0.02 | 0.96 | 0.36 |  | -0.23 | -0.50 |  |  |  |  |  |
| GAAT1F09P | 0.10 | 0.10 | 0.04 |  | -0.09 | -0.06 |  | 0.43 |  |  |  |
| TAGA006 | 0.30 | -0.17 |  | -0.14 |  |  | 0.23 |  |  |  |  |
| GATA30B11 | 0.16 | 0.45 | 0.04 | -1.27 | 1.85 | -0.23 | -1.76 | 2.94 | 0.99 | -0.93 | -0.81 |
| GATA135C03M | 0.25 |  |  |  |  |  |  |  |  |  |  |
| GATA150B10 | 0.20 | 0.13 | -0.09 | 1.47 | -5.47 |  | 0.27 |  |  | 0.35 | 2.33 |
| ATA44F05P | 0.21 | 0.10 | -0.02 | -0.51 | 0.38 | -0.07 |  |  | 0.57 | -0.40 |  |
| GATA138G03 | 0.22 | -0.02 |  | -0.32 | 2.65 |  |  | -1.35 |  |  | -1.47 |
| TAGA049 | 0.30 | 0.73 |  |  |  |  |  |  |  |  |  |
| AATA045 | 0.27 | 0.42 | -0.13 |  | -0.89 |  |  | 1.37 |  |  |  |
| ATT077P | 0.48 | 0.28 |  | -0.48 |  |  | 0.73 |  |  |  |  |
| GATA129D03M | 0.20 | 0.62 |  | 0.18 |  |  | -1.03 |  |  |  |  |
| AGAT099P | 0.14 |  | 0.07 | 0.33 | -0.35 |  |  |  | -0.33 | 0.29 |  |
| GATA31H11P | 0.08 | 0.17 | 0.11 | 1.66 | -2.97 |  |  | -0.95 | -0.89 | 0.99 | 1.28 |
| AAAT111 | -0.15 | 0.95 | 0.17 |  | -0.11 | -0.16 |  |  |  |  |  |
| AAT248P | 0.31 |  |  |  |  |  |  |  |  |  |  |
| ATAG078P | 0.59 | 0.00 | -0.16 | -2.90 | 5.11 |  | -1.43 | 2.90 | 1.72 | -1.44 | -2.99 |
| GATA135G01 | 0.15 | 0.17 | -0.02 | 0.73 | -0.71 |  | -1.75 | 2.07 | 0.14 |  |  |
| GATA12A08P | 0.28 | -0.28 | -0.05 |  | 0.09 | 0.22 |  |  |  |  |  |
| GATA63C02 | 0.41 |  |  |  |  |  |  |  |  |  |  |
| ATAG022 | 0.08 |  | 0.09 | 0.15 | -0.18 |  |  |  | -0.27 | 0.22 |  |
| AGAT030P | 0.27 | 0.24 |  |  |  |  |  |  |  |  |  |
| GATA141B10M | 0.26 | -0.01 | -0.03 | -0.96 | 1.84 |  |  | -1.73 | 0.78 | -0.88 |  |
| GATA138B05 | 0.31 | -0.08 | -0.07 | -0.72 | 0.89 | 0.05 |  |  | 0.34 | -0.42 |  |
| TAGA010 | 0.16 | 0.19 | 0.06 | -0.64 | 1.62 |  | -0.46 |  |  |  | -0.72 |
| GATA142H05P | 0.19 | -0.08 | 0.06 | -0.62 | -0.68 |  | 2.41 | -2.82 | -0.40 | 0.56 | 1.38 |
| GATA51A07P | 0.25 | -0.13 | -0.03 | 0.07 |  | 0.19 |  |  | -0.18 |  |  |
| GATA12G02 | 0.04 | 1.54 | 0.32 | -1.82 | 0.95 | -0.34 | 3.05 | -1.78 |  |  |  |
| AGAT122 | 0.09 | 0.16 | 0.05 | 0.62 | -0.32 |  | -1.56 | 0.86 |  |  |  |
| GATA73D11P | 0.11 | 0.28 | 0.06 | 0.54 | -0.38 |  | -1.48 | 1.44 |  |  |  |
| AAT255 | 0.26 | 0.07 |  |  |  |  |  |  |  |  |  |
| AGAT130 | 0.17 | 0.47 | 0.13 |  |  | -0.20 |  |  |  |  |  |
| GATA51D11P | 0.31 | -0.18 | -0.07 | -0.71 | 1.67 | 0.13 |  |  |  |  | -0.84 |
| ATA092P | 0.28 | -0.04 | 0.01 | -1.37 | 3.89 |  | 0.68 |  |  | -0.21 | -1.91 |
| GATA139B09P | 0.15 | 0.11 | 0.06 | 0.84 | -0.71 |  |  |  | -0.57 | 0.44 |  |
| AGAT126 | 0.25 | 1.00 |  | 1.01 | -0.74 |  | -5.02 | 3.46 |  |  |  |
| AAT266 | 0.33 | 0.41 | -0.06 | -0.32 |  | 0.13 | 0.81 |  |  |  |  |
| AAT013 | 0.13 | 0.12 |  | 0.28 |  |  | -0.36 |  |  |  |  |
| AAAT072 | 0.39 | -0.26 | -0.06 | -0.46 | -0.50 | 0.21 |  | 1.04 | 0.20 |  |  |
| ATT079P | 0.46 | -0.14 |  | -0.60 | 0.49 |  |  |  |  |  |  |
| ATA109H09 | 0.26 | -0.03 | -0.09 |  |  | 0.13 |  |  |  |  |  |
| TTA032Z | 0.14 | 0.38 |  |  |  |  |  |  |  |  |  |
| ATTT030 | -0.09 | 0.67 | 0.11 | 2.03 | -0.60 |  | -3.57 | 2.28 | -0.85 | 0.81 | -0.79 |
| AAT256P | 0.08 | 0.14 | 0.05 | 1.03 | -2.31 |  |  |  |  |  | 1.10 |
| ATA50C05 | 0.30 | 0.05 | -0.11 | -0.84 | 0.62 |  | -1.06 | 1.58 | 1.52 | -1.36 |  |
| ATA12D05P | 0.20 |  | 0.16 | 1.40 | -1.09 |  |  |  | -0.85 | 0.65 |  |
| GATA129G03P | 0.23 | -0.05 | 0.03 | -0.45 | 0.61 |  | 0.35 | -0.38 | -0.07 |  |  |
| ATC033 | 0.37 | 0.19 |  |  |  |  |  |  |  |  |  |
| TAAA014P | 0.10 | 0.47 | 0.13 | 0.95 | -0.70 | -0.28 |  |  |  |  |  |
| GATA29C09P | 0.20 | 0.08 |  |  |  |  |  |  |  |  |  |
| GATA11B08P | 0.34 | 0.01 | -0.08 | -1.14 | 1.96 |  |  | 0.11 | 0.46 | -0.45 | -0.80 |
| GATA112F02P | 0.32 |  |  |  |  |  |  |  |  |  |  |
| GATA30A08M | 0.09 | 1.76 |  |  |  |  |  |  |  |  |  |
| GATA136F05P | 0.63 | 0.21 |  | -1.51 | 2.96 |  |  |  |  |  | -1.45 |
| GATA161A04P | 0.24 | -0.09 | 0.02 | -0.99 | 2.04 |  | 0.88 | -0.49 |  | -0.08 | -0.90 |
| ATA38D08P | 0.19 | 0.78 |  |  |  |  |  |  |  |  |  |
| ATA1F08 | 0.28 | 0.42 | -0.02 | -1.85 | 3.19 |  |  |  | 0.47 | -0.42 | -1.22 |
| TATC050ZM | 0.11 | 0.43 | 0.22 | 1.06 | -0.07 | -0.29 |  | -1.66 | -0.31 |  |  |
| ATA6C09P | 0.48 |  |  | -0.98 | 2.46 |  |  |  |  |  | -1.30 |
| TAT026P | 0.23 |  |  |  |  |  |  |  |  |  |  |
| ATGA020 | 0.38 | 0.26 | -0.22 | 1.36 | -0.75 | 0.37 | -3.78 | 2.08 |  |  |  |
| AGC001B | 0.19 | 0.08 | 0.23 | 0.13 |  | -0.08 |  |  | -0.17 |  |  |
| GATA61G06 | 0.22 | 0.02 | -0.12 | -0.03 | 0.56 | 0.08 | -0.59 |  | 0.53 | -0.47 |  |
| TATC010P | 0.10 | 0.00 | 0.12 | 1.49 | -1.49 |  |  | 0.54 | -0.83 | 0.70 |  |
| TATT019 | 0.20 |  | 0.05 |  |  |  |  |  |  |  |  |
| GATA137A12M | 0.11 | 0.11 |  | 0.33 | -0.26 |  |  |  |  |  |  |
| AGAT103 | 0.07 | 0.29 | -0.04 | 1.77 | -2.70 |  | -1.16 | 0.95 |  |  | 0.97 |
| GATA026M | 0.25 | -0.04 | -0.03 | -0.35 | 2.27 | 0.11 | -1.03 | 1.21 |  | -0.11 | -1.58 |
| ATA31F09M | 0.44 | -0.03 |  | -2.45 | 3.54 |  | 2.35 | -1.38 |  |  | -1.41 |
| TAT028P | 0.28 | -0.05 |  | -0.50 | 0.39 |  | 0.39 | -0.29 |  |  |  |
| GATA4E04 | 0.28 | 0.11 |  |  |  |  |  |  |  |  |  |
| GATA21D12P | 0.12 | 0.57 | 0.15 |  | -0.05 | -0.40 |  | 0.21 |  |  |  |
| GATA87D11 | 0.21 |  |  |  |  |  |  |  |  |  |  |
| ATA78C09ZP | 0.23 | 0.42 | 0.14 |  | -0.14 | -0.23 |  | 0.49 |  |  |  |
| ATAC037P | 0.41 |  | 0.10 |  |  |  |  |  |  |  |  |
| TTTA001M | 0.02 | 0.53 | 0.12 | 3.09 | -4.90 |  | -1.74 | 1.99 | -1.16 | 1.12 | 1.27 |
| AGAT133 | 0.83 | -0.67 | -0.19 | -0.05 | 0.52 | 0.46 | 2.91 | -3.67 | -1.68 | 1.42 |  |
| ATA55A05 | 0.31 | -0.03 | 0.00 | -1.00 | 0.66 |  |  | 0.23 | 0.28 | -0.23 |  |
| GATA145G10M | 0.19 | 0.17 |  |  |  |  |  |  |  |  |  |
| GATA63F08P | 0.36 |  |  | -0.19 |  |  |  |  |  |  |  |
| AGAT049P | 0.80 | -0.08 | -0.31 |  |  | 0.51 |  |  |  |  |  |
| TATG002P | 0.12 | 0.18 | 0.11 | -0.13 |  | -0.16 | 0.93 |  | -0.11 |  |  |
| MFD442-GTTT002 | 0.09 | 0.15 | 0.06 | -0.80 | 1.23 |  | 3.35 | -3.05 | -0.15 |  |  |
| ATT023 | 0.22 | 0.27 | -0.09 | -0.70 | 1.41 |  |  | -0.62 | 0.64 | -0.70 |  |
| ATAA009 | 0.29 | 0.10 |  |  |  |  |  |  |  |  |  |
| TTCA004P | 0.20 |  | 0.07 | 0.47 | -0.29 |  |  |  | -0.42 | 0.31 |  |
| GATA25C10M | 0.22 |  | 0.23 | 0.72 | -0.60 |  |  |  | -0.96 | 0.69 |  |
| ATT070 | 0.42 |  |  | 0.68 | -0.55 |  |  |  |  |  |  |
| TATC012 | 0.17 | 0.32 | 0.01 | 0.12 | -0.01 |  |  | -0.29 | -0.52 | 0.46 |  |
| ATAA018P | 0.10 | 0.15 | 0.12 | -0.63 | 2.14 | -0.14 | 2.37 | -2.62 | -0.55 | 0.38 | -1.07 |
| TTA024 | 0.09 | 0.15 | 0.02 |  | 0.02 | -0.05 |  | -0.06 |  |  |  |
| AATA019 | 0.24 |  | 0.12 | 0.32 | -0.24 |  |  |  | -0.54 | 0.37 |  |
| GATA156H01M | 0.43 | 0.15 | -0.02 | 1.56 | -0.94 |  | -2.38 | 2.60 | -0.21 |  |  |
| ATAG042 | 0.32 | -0.05 |  | -1.28 | 1.96 |  |  | 0.44 |  |  | -0.90 |
| AAT076 | 0.15 | -0.01 | 0.30 | 0.76 | -0.70 | -0.18 | 1.53 | -1.56 | -1.30 | 1.26 |  |
| GATA3H11 | 0.26 |  | -0.03 | -0.67 | 0.55 |  |  |  | 0.31 | -0.27 |  |
| AGAT115 | 0.28 | 0.69 |  | -0.48 | 0.54 |  | 3.92 | -3.34 |  |  |  |
| AAAT121P | 0.42 | 0.45 | -0.01 | -1.34 | 2.74 | 0.27 | 3.61 | -3.91 | -1.07 | 0.92 | -1.14 |
| TATC059 | 0.28 |  |  |  |  |  |  |  |  |  |  |
| GATA060 | 0.56 | -0.47 | -0.04 | 0.42 | -0.34 | 0.31 |  |  | -0.70 | 0.44 |  |
| AAT052 | 0.46 |  |  |  |  |  |  |  |  |  |  |
| TAA005 | 0.12 |  | 0.03 | -0.20 | 1.26 |  |  |  | -0.12 |  | -0.69 |
| AAAAC001 | 0.17 | 0.18 | 0.20 | 1.82 | -4.11 |  |  | 3.51 | -1.13 | 1.06 | 1.37 |
| GATA175H06M | 0.32 | -0.08 |  | -1.41 | 2.42 |  | 2.19 | -3.06 |  |  | -0.58 |
| AGAT142P | 0.21 |  | 0.11 | -1.16 | 3.04 |  |  |  |  | -0.13 | -1.43 |
| TCTA020 | 0.35 | -0.17 | -0.07 |  |  | 0.11 |  |  |  |  |  |
| GATA165A11M | 0.20 | 0.00 | 0.16 | 0.62 | 0.00 |  |  | -0.32 | -0.94 | 0.71 |  |
| ATA65H08P | 0.42 |  |  |  |  |  |  |  |  |  |  |
| ATA18C09P | 0.16 | 0.40 |  | -0.68 | 1.81 |  |  | 0.30 |  |  | -1.03 |
| GATA5E06P | 0.35 | 1.17 |  | 0.39 | -0.51 |  | -2.48 | 2.06 |  |  |  |
| GATA61F04 | 0.28 | 1.61 |  |  |  |  |  |  |  |  |  |
| AGAT140P | 0.24 | 0.12 | 0.02 |  |  | 0.10 |  |  |  |  |  |
| GATA22H04M | 0.16 | 0.18 | -0.01 | 1.28 | -1.50 |  | -1.49 | 1.64 |  | 0.10 |  |
| GGAT3G09M | 0.34 | 0.42 |  | -0.21 |  |  | 0.83 |  |  |  |  |
| AAT261 | 0.22 | 0.46 |  |  |  |  |  |  |  |  |  |
| AAAT126 | 0.14 | 0.50 | -0.11 | 0.15 |  | 0.22 |  |  |  |  |  |
| GATA27Z | 0.10 | 0.25 | 0.12 | -1.41 | 3.39 | -0.10 |  | -0.67 | 0.29 | -0.26 | -1.48 |
| ATA42G04P | 0.12 | 0.82 | 0.06 | -0.15 |  | -0.24 |  |  |  |  |  |
| GATA65D11 | 0.34 | -0.05 | -0.08 | 0.25 | -0.11 | 0.23 | -2.52 | 1.53 |  |  |  |
| TCTA017M | 0.29 |  |  |  | -0.09 |  |  |  |  |  |  |
| AGAT125 | 0.14 | 0.83 |  |  |  |  |  |  |  |  |  |
| CTAT016 | 0.17 | -0.03 | 0.05 | 0.08 | 0.43 |  | 0.36 |  | -0.56 | 0.49 | -0.73 |
| TTTTA002 | 0.03 | 0.18 | 0.29 | 1.36 | -0.53 |  |  | -0.81 | -1.43 | 1.19 |  |
| ATAG053P | 0.49 |  |  |  |  |  |  |  |  |  |  |
| ATA84D02 | 0.06 | 0.54 | 0.05 |  |  |  |  |  |  |  |  |
| ATCC001 | 0.11 | -0.03 | 0.01 | 0.74 | -0.55 | 0.05 |  |  | -0.30 | 0.22 |  |
| ATGT009P | 0.22 | 0.64 |  |  |  |  |  |  |  |  |  |
| ATAG055 | 0.24 | 0.24 |  |  |  |  |  |  |  |  |  |
| GATA179E06P | 0.27 | -0.01 | -0.06 | 1.43 | -2.97 |  | 1.19 | -0.89 |  |  | 1.44 |
| ATA20B07 | -0.01 | 0.35 |  |  |  |  |  |  |  |  |  |
| TCTA021ZM | 0.03 | 0.21 | 0.05 | 1.28 | -2.29 |  |  |  |  |  | 0.94 |
| GATA90G05P | 0.29 |  |  | -0.85 | 1.70 |  |  |  |  |  | -0.76 |
| GATA196C10P | 0.15 | 0.28 |  | 1.61 | -3.58 |  | -1.29 | 1.66 |  |  | 1.53 |
| GATA134F03P | -0.04 | 0.98 | 0.14 | 0.98 | -2.12 | -0.59 |  |  |  |  | 0.96 |
| GATA81F06 | 0.21 | 0.20 | -0.05 | -0.51 | 0.35 |  |  |  | 0.56 | -0.45 |  |
| ATA80B10Z | 0.30 | 0.62 |  | -0.32 |  |  |  |  |  |  |  |
| TTA007M | 0.04 | 0.19 | 0.13 | 0.94 | -0.58 |  |  | -0.78 | -0.69 | 0.63 |  |
| GATA88F08P | 0.17 | 0.13 | 0.05 | -0.31 | 0.93 |  | 1.47 | -2.55 |  | -0.14 |  |
| ATA44G07M | 0.26 | -0.06 | 0.11 | 0.69 | -2.10 |  | 0.91 | -0.62 |  |  | 1.02 |
| AAC1C02 | 0.20 | 0.11 |  | -0.12 |  |  |  |  |  |  |  |
| TACA003 | 0.06 | 0.06 | 0.03 | 0.82 | -0.60 |  |  |  | -0.34 | 0.26 |  |
| ATGT006Z | 0.33 |  |  |  |  |  |  |  |  |  |  |
| AAT224M | 0.23 | 0.51 |  | -0.25 |  |  |  |  |  |  |  |
| GATA73B08M | 0.17 | 0.53 | 0.27 | 2.33 | -1.81 |  | -5.14 | 5.86 | -1.38 | 0.94 |  |
| ATA25D12 | 0.72 | -0.23 | -0.11 | 0.42 |  | 0.20 |  |  | -0.28 |  |  |
| GATA29B11 | 0.79 | -0.07 | -0.19 | -3.48 | 5.40 |  | 1.59 |  | 1.08 | -1.25 | -2.27 |
| AAT268 | 0.45 | -0.15 | -0.09 | -0.20 |  | 0.15 |  |  |  |  |  |
| TTA008P | 0.18 | -0.09 | 0.15 | -0.39 | 0.58 |  | 2.49 | -2.85 | -1.08 | 1.01 |  |
| AAT265M | 0.14 | 0.52 | 0.00 | -0.25 | 1.60 | 0.15 |  |  | -0.16 |  | -0.90 |
| TCTA025 | 0.20 | 0.24 | 0.05 | -0.80 | 2.19 |  |  |  | -0.16 |  | -1.08 |
| GATA35 | 0.43 | 0.27 | 0.15 | -1.76 | 3.26 | -0.14 |  |  |  |  | -1.49 |
| GATA71E06 | 0.19 | 0.18 | 0.03 | 0.48 | -0.42 |  |  |  | -0.43 | 0.35 |  |
| AAT228 | 0.33 | -0.02 |  | -1.00 | 1.07 |  | 0.77 | -0.85 |  |  |  |
| TAA003 | 0.17 | 0.27 |  |  |  |  |  |  |  |  |  |
| ATA27C11 | 0.07 | 0.39 | -0.04 | 0.80 | -0.77 | 0.10 | -0.91 | 1.04 |  |  |  |
| GATA101G01 | 0.26 |  | 0.14 | 2.07 | -1.44 |  |  |  | -1.16 | 0.85 |  |
| GATA22H02 | 0.04 | 0.87 | 0.10 | 0.29 | -0.12 | -0.28 | -1.53 | 0.92 |  |  |  |
| AAT262 | 0.12 | 1.55 |  |  |  |  |  |  |  |  |  |
| GATA7F09 | 0.22 | 0.12 |  |  |  |  |  |  |  |  |  |
| ATAAC002 | 0.18 | -0.08 | 0.06 | 1.41 | -0.51 | 0.26 | -0.95 |  | -0.91 | 0.70 |  |
| AAC040Z | 0.13 |  | 0.28 | 0.54 | -0.39 |  |  |  | -1.15 | 0.83 |  |
| GATA91H01 | 0.26 | 0.23 | 0.11 | 2.07 | -2.10 |  | -2.48 | 2.46 | -1.02 | 0.95 |  |
| AAT142M | 0.12 | 0.63 | 0.10 | 0.26 | 0.30 |  | -2.23 | 3.07 | -0.95 | 1.02 | -1.30 |
| ATA73C05P | 0.34 | 0.26 | -0.06 | -1.03 | 0.83 |  |  |  | 0.51 | -0.46 |  |
| GATA167C12 | 0.15 | 0.01 | 0.05 | 1.32 | -1.90 |  | -0.29 |  | -0.46 | 0.43 | 0.79 |
| AGAT084 | 0.11 | 0.09 | 0.05 | 0.12 |  |  |  |  |  |  |  |
| GGAT2G06M | 0.04 | 0.43 |  |  |  |  |  |  |  |  |  |
| AGAT114 | -0.06 | 0.64 | 0.19 | 2.72 | -3.13 |  | -2.73 | 3.84 | -1.16 | 1.03 |  |
| ATA10H03 | 0.31 | 0.04 | -0.05 | -0.95 | 0.70 |  | 2.02 | -1.58 |  |  |  |
| GGAA19H02 | 0.37 | -0.11 | -0.07 |  |  | 0.18 |  |  |  |  |  |
| ATA63A05P | 0.10 | 0.68 | 0.04 | -0.15 |  | -0.10 | 0.64 |  |  |  |  |
| GGAA22C05 | 0.46 | -0.10 | -0.05 | -0.74 | 1.89 |  | 0.71 |  |  | -0.15 | -1.08 |
| GATA5H03 | 0.09 | 0.50 | 0.07 | -0.33 | -0.18 | -0.15 |  | 0.32 |  | 0.10 |  |
| ATA080M | 0.15 | 0.21 | 0.10 | -0.17 | -0.02 |  | 1.41 | -1.40 | -0.77 | 0.68 |  |
| AAT253P | 0.12 | 0.07 | 0.04 | 0.34 | -0.29 |  |  |  | -0.30 | 0.24 |  |
| AAAAT002 | 0.17 | 0.25 |  | 0.89 | -1.53 |  |  | 1.08 |  |  |  |
| AAAT134 | 0.27 | -0.06 | -0.08 | 0.39 | -0.89 | 0.09 | -0.64 | 1.04 |  | 0.14 |  |
| GATA86B09P | 0.22 |  |  |  |  |  |  |  |  |  |  |
| AGAT110P | 0.37 | 0.63 | 0.10 | -0.35 |  |  |  |  |  |  |  |
| GATA6B07 | 0.16 | 0.19 | 0.10 | 0.18 | 1.25 |  |  |  | -0.73 | 0.60 | -0.94 |
| GATA73A05 | 0.23 |  |  |  |  |  |  |  |  |  |  |
| AACAT001 | 0.47 |  |  |  |  |  |  |  |  |  |  |
| GATA137B09 | 0.07 | 0.20 | 0.20 | -0.86 | 1.87 | -0.08 |  |  | -0.44 | 0.32 | -0.91 |
| ATA38B10 | 0.32 |  | 0.04 | -0.56 | 0.37 |  |  |  |  |  |  |
| ACT3F12 | 0.32 | 0.33 | -0.11 |  | -0.07 |  |  | -0.43 |  | 0.12 |  |
| GATA100E02P | 0.20 | 0.00 | -0.03 | -0.84 | 0.87 |  | 0.87 | -0.76 | 0.47 | -0.44 |  |
| GATA135E01P | 0.20 | 0.03 | 0.18 | 0.04 |  | -0.05 |  |  | -0.15 |  |  |
| AGAT113Z | 0.34 | 0.09 |  |  | -0.13 |  |  |  |  |  |  |
| GTT035 | 0.35 | 0.15 |  | 1.26 | -1.15 |  | -2.17 | 2.27 |  |  |  |
| ATA70B03P | 0.38 | -0.03 | -0.07 | -1.05 | 2.61 | 0.17 |  |  |  |  | -1.26 |
| ATA77F05 | 0.21 | 0.04 | 0.06 | -0.46 | 0.27 |  | 3.53 | -2.57 |  |  |  |
| TCTA023P | 0.23 | 0.18 | -0.06 | -1.02 | 1.69 |  | -0.43 |  | 0.51 | -0.40 | -0.52 |
| AGAT116P | 0.47 | -0.09 | -0.02 |  | -0.60 |  |  | 1.28 |  | 0.11 |  |
| GATA136A04 | 0.36 | -0.04 |  | -0.89 | 0.83 |  | 3.80 | -3.50 |  |  |  |
| GATA90G11M | 0.19 |  |  |  |  |  |  |  |  |  |  |
| GGAA30H04 | 0.12 | 1.12 |  | 0.13 |  |  | -0.81 |  |  |  |  |
| AGAT131 | 0.21 | 0.93 | 0.46 | -2.76 | 1.67 | -0.39 | 4.14 | -2.70 |  |  |  |
| ATA069P | 0.19 | 0.04 | 0.10 | 0.12 | -0.06 |  | 1.90 | -2.44 | -0.53 | 0.49 |  |
| GATA169E06 | 0.15 | 0.00 | 0.08 |  | -0.14 |  |  | 0.53 |  |  |  |
| ATAC026P | 0.73 |  |  | 2.42 | -1.84 |  |  |  |  |  |  |
| GATA91G06 | 0.39 |  |  | -0.18 |  |  |  |  |  |  |  |
| GATA51F04P | 0.25 |  |  |  |  |  |  |  |  |  |  |
| GATA045 | 0.26 | 0.33 |  | 1.08 | -2.19 |  |  |  |  |  | 0.93 |
| ATGG002 | 0.11 | 0.37 |  | -0.70 | 0.74 |  |  |  |  |  |  |
| ATT198Z | 0.23 | 0.18 |  |  | -0.13 |  |  |  |  |  |  |
| GATA143C02 | 0.26 |  |  | -0.47 | 1.20 |  |  |  |  |  | -0.63 |
| GAAA1C11 | 0.13 | 1.38 |  | 2.58 | -4.78 |  | -6.48 | 6.51 |  |  | 2.01 |
| TAT032Z | 0.46 | -0.11 | -0.23 | 0.12 | 0.43 | 0.18 | -1.02 |  | 0.81 | -0.65 |  |
| GATA153F11 | 0.22 | 0.27 |  | -0.78 | 0.60 |  |  | 0.96 |  |  |  |
| AATA053 | 0.13 |  |  | 0.34 | -0.22 |  |  |  |  |  |  |
| GATA63B12P | 0.50 |  |  |  |  |  |  |  |  |  |  |
| TTAT027P | 0.36 | 0.53 |  |  |  |  |  |  |  |  |  |
| AGAT073P | 0.16 | 0.17 | 0.05 | 0.87 | -2.28 |  |  |  |  |  | 1.16 |
| GATA22F01 | 0.13 | 0.27 | 0.06 | 0.16 |  |  |  |  |  |  |  |
| TAGA031Z | 0.09 | 0.18 | 0.07 | 0.32 | 2.23 |  |  | -0.41 |  | -0.19 | -1.29 |
| TTTA028 | 0.37 | 0.13 | -0.02 | -0.65 | -3.42 |  | 1.39 | -1.19 | 0.36 |  | 2.48 |
| ATA67B07P | 0.02 | -0.01 | 0.15 | -0.01 | 0.16 |  | 0.57 | -0.57 | -0.64 | 0.47 |  |
| GATA5H07M | 0.21 | -0.02 | 0.05 |  | -0.14 |  |  | 0.29 |  |  |  |
| GATA86C08P | 0.34 | -0.04 | -0.12 | 0.12 |  | 0.18 | -0.46 |  |  |  |  |
| ATA10H10P | 0.14 | 0.83 | 0.05 | -0.37 | -0.22 | -0.40 | -1.47 | 3.29 | 0.43 | -0.40 |  |
| AAT226 | 0.11 | 0.66 |  |  | -0.04 |  |  | 0.52 |  |  |  |
| TTAT023Z | 0.43 |  |  |  |  |  |  |  |  |  |  |
| CATA002Z | 0.19 | 0.02 | -0.04 |  |  | 0.05 |  |  |  |  |  |
| GATA143D05 | 0.17 | 0.04 | 0.06 |  | 0.49 |  |  | -0.42 |  | -0.12 |  |
| GATA151C03P | 0.20 |  |  |  | -0.09 |  |  |  |  |  |  |
| ATA103C03P | 0.07 | 0.74 |  |  |  |  |  |  |  |  |  |
| AAT107 | 0.14 | 0.08 | -0.05 | 0.97 | -0.69 |  | -0.30 |  | 0.15 |  |  |
| GATA140E03 | 0.10 | 0.36 | 0.14 |  |  | -0.24 |  |  |  |  |  |
| TCTA026 | 0.30 | -0.10 | -0.01 | -0.66 | 0.45 |  | 1.52 | -1.20 |  | 0.09 |  |
| MFD466 | 0.11 | 0.66 | 0.08 |  |  | -0.19 |  |  |  |  |  |
| GATG013M | 0.22 | 0.15 |  |  |  |  |  |  |  |  |  |
| ATACC001 | 0.00 | 0.32 | 0.30 | 1.16 | -0.87 |  |  |  | -1.18 | 0.77 |  |
| ATA063 | 0.17 | 0.56 | -0.07 | 0.12 |  |  |  |  |  |  |  |
| CTAT003 | 0.13 | 0.16 |  |  |  |  |  |  |  |  |  |
| GATA158H04 | 0.05 | 0.55 | 0.00 | 1.84 | -2.95 |  | -3.64 | 5.41 |  | -0.37 | 1.24 |
| AGAT132 | 0.17 | 0.54 |  |  |  |  |  |  |  |  |  |
| GATA64B04P | 0.12 | 0.50 |  |  |  |  |  |  |  |  |  |
| GATA10H07P | 0.24 | 0.19 | -0.10 | 0.28 |  | 0.21 | -0.67 |  |  |  |  |
| AAT083 | 0.07 | 0.93 | 0.03 | -0.89 | 3.47 | -0.10 |  | 0.66 |  | -0.27 | -1.98 |
| ATA58E08ZP | 0.48 | 0.12 | -0.14 | 2.03 | -5.05 | 0.25 |  |  |  | 0.14 | 2.21 |
| GATA169F02 | 0.17 |  | 0.22 | 0.51 | -0.51 |  |  |  | -0.73 | 0.49 |  |
| GGAA19G04 | 0.65 | 0.60 |  |  |  |  |  |  |  |  |  |
| AAT245 | 0.02 | 0.54 | 0.22 | 0.65 | 0.65 |  |  | -0.68 | -0.31 |  |  |
| GATA31B11 | 0.32 | -0.11 | 0.04 | -1.36 | 2.33 |  | 0.72 | -0.90 |  | -0.11 | -0.65 |
| GATA63G01 | 0.21 | 0.57 | -0.10 | 0.84 | -2.19 |  | -0.56 |  | 0.20 |  | 0.99 |
| TTCA006M | 0.13 | 0.16 | 0.08 | -0.21 |  |  |  |  |  |  |  |
| AAT095 | 0.33 | 0.01 |  | -0.56 | 0.43 |  | 2.01 | -1.35 |  |  |  |
| CTG008 | 0.12 | 0.02 | 0.15 | 0.87 | -0.30 | 0.49 |  | -1.16 | -1.35 | 1.04 |  |
| ATCT050 | 0.30 |  |  |  |  |  |  |  |  |  |  |
| AGAT060 | 0.33 | 0.03 | -0.10 |  |  | 0.24 |  |  |  |  |  |
| GATA036 | 0.33 |  | 0.10 | -0.28 | 3.24 |  |  |  | -0.40 |  | -1.79 |
| AGAT127 | 0.19 | 0.12 |  |  |  |  |  |  |  |  |  |
| GATA062 | 0.25 |  |  |  |  |  |  |  |  |  |  |
| GATA85D10 | 0.24 | -0.05 |  | -0.37 | 0.19 |  | 0.94 | -0.54 |  |  |  |
| GATA183H03Z | 0.26 | 0.16 | 0.07 | -1.28 | 2.78 |  |  |  | -0.16 |  | -1.14 |
| GATA173A03 | 0.27 | -0.19 | 0.32 | 0.99 | -0.53 |  |  |  | -1.03 | 0.59 |  |
| GATA73D05 | 0.50 |  |  | -0.25 |  |  |  |  |  |  |  |
| ATAG089P | 0.31 | -0.06 | -0.03 | -0.68 | 0.48 |  | 0.51 | -0.37 |  |  |  |
| GATA129F05 | 0.23 |  |  | -0.51 | 1.22 |  |  |  |  |  | -0.56 |
| GATA157H01 | 0.02 | 0.33 | 0.21 | 1.06 | -1.51 | -0.16 | -2.04 | 2.37 |  |  |  |
| AGAT138P | 0.91 |  |  | -2.82 | 5.99 |  |  |  |  |  | -2.78 |
| GATA146H09 | 0.41 | 0.28 | 0.18 | 0.45 | -0.74 | -0.19 | 0.71 |  |  |  |  |
| TGA012P | 0.19 |  | -0.08 | -0.37 | 0.23 |  |  |  | 0.45 | -0.29 |  |
| GGAA21A04 | 0.25 |  | 0.10 |  |  |  |  |  |  |  |  |
| GATA156F11 | 0.07 | 0.29 | -0.02 | 1.24 | -2.93 |  | -1.26 | 2.06 |  | 0.07 | 1.09 |
| AAT260M | 0.48 | -0.13 | -0.16 |  |  | 0.26 |  |  |  |  |  |
| AAT257 | 0.25 | 0.10 | 0.12 | -1.30 | 3.01 | -0.16 | 2.18 | -1.57 |  | -0.21 | -1.22 |
| TTTA075P | 0.48 | 0.18 | -0.09 |  |  |  |  |  |  |  |  |
| AAT249 | 0.25 | 0.25 |  |  |  |  |  |  |  |  |  |
| AAAT007 | 0.15 | 0.19 | 0.16 |  |  | -0.14 |  |  |  |  |  |
| AAT247 | 0.06 | 0.12 | 0.10 | 0.20 | -0.02 | -0.10 | -0.19 |  |  | 0.17 |  |
| AGAT136M | 0.12 | 0.67 | 0.13 | 0.57 | -0.64 |  |  |  | -0.71 | 0.60 |  |
| GATA72E11 | 0.23 | 0.56 | 0.16 | -2.94 | 8.14 | -0.29 |  | -1.77 |  | -0.36 | -3.61 |
| ATTC013 | 0.20 | 0.50 |  | -0.64 | 3.29 |  |  | -0.85 |  |  | -1.82 |
| GATA81E09 | 0.29 |  | 0.10 |  |  |  |  |  |  |  |  |
| AGAT139P | 0.12 | 0.10 | -0.05 | -0.12 | 0.59 |  |  | -0.42 | 0.36 | -0.42 |  |
| GATA142C02M | 0.22 |  | 0.16 | -0.27 | 1.81 |  |  |  | -0.81 | 0.49 | -1.00 |
| GATA65E01 | 0.35 | 0.00 | 0.24 | -2.47 | 4.63 |  | 2.89 |  | -0.42 |  | -2.18 |
| GATA90E02 | 0.33 | 0.43 |  |  |  |  |  |  |  |  |  |
| ATCT035 | 0.12 | 0.18 | 0.10 | 0.20 |  | -0.12 | -0.52 |  |  |  |  |
| AAT269 | 0.25 | 0.11 |  |  | -0.35 |  |  | 1.24 |  |  |  |
| TATT031 | 0.45 |  |  |  |  |  |  |  |  |  |  |
| TTTA093P | 0.39 |  |  |  |  |  |  |  |  |  |  |
| GATA163G03 | 0.13 |  | 0.12 | 1.04 | -0.74 |  |  |  | -0.72 | 0.51 |  |
| TATC057 | 0.10 | 0.83 |  | 0.51 | -1.49 |  |  |  |  |  | 0.82 |
| GATA148F04P | 0.32 | 0.00 |  | -0.43 | 0.53 |  |  | -0.53 |  |  |  |
| AGAT057 | 0.42 |  | -0.08 | 0.91 | -0.68 |  |  |  |  |  |  |
| AGAT120 | 0.11 | 0.20 | 0.01 | 0.16 | 1.04 |  | -1.37 | 1.50 |  | -0.11 | -1.09 |
| TTA015P | 0.18 | 0.34 | -0.06 |  |  | 0.14 |  |  |  |  |  |
| ATTT019M | -0.05 | 0.20 | 0.11 | 0.82 | -0.50 |  | -0.76 | 0.44 | -0.07 |  |  |
| AGAT055Z | 0.45 | 0.05 | -0.23 | 0.95 | -0.92 | 0.46 |  |  |  |  |  |
| GATA6F05P | 0.27 |  | 0.06 | -0.21 |  |  |  |  |  |  |  |
| TCAT006ZP | 0.18 |  |  |  |  |  |  |  |  |  |  |
| GTAT005Z | 0.14 | 0.41 |  | -0.68 | 1.54 |  |  |  |  |  | -0.75 |
| TTAT020P | 0.19 |  |  |  |  |  |  |  |  |  |  |
| SCA10 | 0.51 |  |  |  |  |  |  |  |  |  |  |
| GATA030P | 0.14 | 0.74 |  |  | 0.43 |  |  | -0.44 |  |  |  |
| TCTA015M | 0.27 |  |  | -0.11 |  |  |  |  |  |  |  |
