# Supplementary material for: Heterozygosity increases microsatellite mutation rate, linking it to demographic history
Source: BMC Genet. 2008 Nov 14;9:72. doi: 10.1186/1471-2156-9-72 (PMC2615044; doi:10.1186/1471-2156-9-72)
Supplement: Additional file 3 — Estimates of modern population size used in the current study [file 1471-2156-9-72-S3.doc]

**Additional File 3:** Population size estimates used in this study. Some obvious anomalies are present. For example the Han Chinese are the largest ethnic group in the world but not in this table. This reflects how explicit the sampling details were for each group, one description of the Han being that they are largely from Guangdong, while the French sample is described as being from ‘several regions in France’. However, the impact of these decisions is likely modest when log(size) is used, as in our analyses. For consistency, all population size estimates were taken from the Joshua project ([http://www.joshuaproject.net](http://www.joshuaproject.net/)).

| **Population** | **Region** | **Size** | **Log Size** |
| --- | --- | --- | --- |
| Papuan | Oceania | 5500000 | 6.74 |
| Melanesian | Oceania | 7000000 | 6.85 |
| Bedouin | Middle East | 1500000 | 6.18 |
| Mozabite | Middle East | 230000 | 5.36 |
| Palestinian | Middle East | 5000000 | 6.70 |
| Druze | Middle East | 200000 | 5.30 |
| Adygei | Europe | 500000 | 5.70 |
| Basque | Europe | 600000 | 5.78 |
| French | Europe | 50000000 | 7.70 |
| Italian | Europe | 58000000 | 7.76 |
| Orcadian | Europe | 19000 | 4.28 |
| Russian | Europe | 143000000 | 8.16 |
| Sardinian | Europe | 1600000 | 6.20 |
| Tuscan | Europe | 3500000 | 6.54 |
| Cambodian | E Asia | 13000000 | 7.11 |
| Dai | E Asia | 1000 | 3.00 |
| Daur | E Asia | 154000 | 5.19 |
| Han | E Asia | 36000000 | 7.56 |
| Han-NChina | E Asia | 40000000 | 7.60 |
| Hezhen | E Asia | 11000 | 4.04 |
| Japanese | E Asia | 126000000 | 8.10 |
| Lahu | E Asia | 711000 | 5.85 |
| Miao | E Asia | 77000 | 4.89 |
| Mongola | E Asia | 132000 | 5.12 |
| Naxi | E Asia | 300000 | 5.48 |
| Oroqen | E Asia | 9200 | 3.96 |
| She | E Asia | 800000 | 5.90 |
| Tu | E Asia | 200000 | 5.30 |
| Tujia | E Asia | 7555000 | 6.88 |
| Uygur | E Asia | 9632000 | 6.98 |
| Xibo | E Asia | 83000 | 4.92 |
| Yakut | E Asia | 423000 | 5.63 |
| Yi | E Asia | 6500000 | 6.81 |
| Balochi | C/S Asia | 4500000 | 6.65 |
| Brahui | C/S Asia | 2300000 | 6.36 |
| Burusho | C/S Asia | 100000 | 5.00 |
| Hazara | C/S Asia | 162000 | 5.21 |
| Kalash | C/S Asia | 3000 | 3.48 |
| Makrani | C/S Asia | 2500000 | 6.40 |
| Pathan | C/S Asia | 12000000 | 7.08 |
| Sindhi | C/S Asia | 30000000 | 7.48 |
| Pima | American | 6500 | 3.81 |
| Surui | American | 1000 | 3.00 |
| Colombian | American | 44000000 | 7.64 |
| Maya | American | 800000 | 5.90 |
| Karitiana | American | 400 | 2.60 |
| BiakaPygmy | Africa | 50000 | 4.70 |
| Mandenka | Africa | 130000 | 5.11 |
| Yoruba | Africa | 24000000 | 7.38 |
| BantuKenya | Africa | 20000000 | 7.30 |
| MbutiPygmy | Africa | 40000 | 4.60 |
| San | Africa | 31000 | 4.49 |
| BantuSAfrica | Africa | 1000000 | 6.00 |
